# Ubiquitin-Mediated Response to Microsporidia and Virus Infection in *C. elegans*

**DOI:** 10.1371/journal.ppat.1004200

**Published:** 2014-06-19

**Authors:** Malina A. Bakowski, Christopher A. Desjardins, Margery G. Smelkinson, Tiffany A. Dunbar, Isaac F. Lopez-Moyado, Scott A. Rifkin, Christina A. Cuomo, Emily R. Troemel

**Affiliations:** 1 Division of Biological Sciences, Section of Cell and Developmental Biology, University of California San Diego, La Jolla, California, United States of America; 2 The Broad Institute of MIT and Harvard, Cambridge, Massachusetts, United States of America; 3 Bioinformatics and Systems Biology Graduate Program, University of California San Diego, La Jolla, California, United States of America; 4 Division of Biological Sciences, Section of Ecology, Behavior, and Evolution University of California San Diego, La Jolla, California, United States of America; Stanford University, United States of America

## Abstract

Microsporidia comprise a phylum of over 1400 species of obligate intracellular pathogens that can infect almost all animals, but little is known about the host response to these parasites. Here we use the whole-animal host *C. elegans* to show an *in vivo* role for ubiquitin-mediated response to the microsporidian species *Nematocida parisii*, as well to the Orsay virus, another natural intracellular pathogen of *C. elegans*. We analyze gene expression of *C. elegans* in response to *N. parisii*, and find that it is similar to response to viral infection. Notably, we find an upregulation of SCF ubiquitin ligase components, such as the cullin ortholog *cul-6*, which we show is important for ubiquitin targeting of *N. parisii* cells in the intestine. We show that ubiquitylation components, the proteasome, and the autophagy pathway are all important for defense against *N. parisii* infection. We also find that SCF ligase components like *cul-6* promote defense against viral infection, where they have a more robust role than against *N. parisii* infection. This difference may be due to suppression of the host ubiquitylation system by *N. parisii*: when *N. parisii* is crippled by anti-microsporidia drugs, the host can more effectively target pathogen cells for ubiquitylation. Intriguingly, inhibition of the ubiquitin-proteasome system (UPS) increases expression of infection-upregulated SCF ligase components, indicating that a trigger for transcriptional response to intracellular infection by *N. parisii* and virus may be perturbation of the UPS. Altogether, our results demonstrate an *in vivo* role for ubiquitin-mediated defense against microsporidian and viral infections in *C. elegans*.

## Author Summary

Microbial pathogens have two distinct lifestyles: some pathogens live outside of host cells, and others live inside of host cells and are called intracellular pathogens. Microsporidia are fungal-related intracellular pathogens that can infect all animals, but are poorly understood. We used the roundworm *C. elegans* as a host to show that ubiquitin pathways provide defense against both a natural microsporidian infection of *C. elegans*, as well as a natural viral infection. Our study shows that ubiquitin, the proteasome and autophagy components are all important to control intracellular infection in *C. elegans*, although microsporidia seem to partially evade this defense. We also show that SCF ubiquitin ligases help control both microsporidia and virus infection. Furthermore, we find that *C. elegans* upregulates expression of SCF ligases when ubiquitin-related degradation machinery is inhibited, indicating that *C. elegans* monitors the functioning of this core cellular process and upregulates ligase expression when it is perturbed. Altogether, our findings describe ubiquitin-mediated pathways that are involved in host response and defense against intracellular pathogens, and how this machinery is regulated by infection to increase defense against intracellular pathogens such as microsporidia and viruses.

## Introduction

The Microsporidia phylum contains over 1400 species of obligate intracellular pathogens most closely related to fungi [1]. These pathogens can infect a wide variety of animal hosts including humans, where they can cause significant disease. Infections in humans can cause lethal diarrhea in immunocompromised people such as AIDS patients, and microsporidia are considered priority pathogens at the National Institutes of Health [2], [3]. Microsporidia can also plague agriculturally significant animals such as fish and honeybees [4], [5], [6]. Treatment options for microsporidia infections are limited and often ineffective [7], [8]. In mammals, studies have shown that T cells and dendritic cells provide protection against infection, but little is known about the innate and/or intracellular responses to these pathogens [9], [10], [11].

Previously, we described *Nematocida parisii*, a microsporidian species isolated from a wild-caught *C. elegans* near Paris, which causes a lethal intestinal infection in its host [12], [13]. *N. parisii* infection of the simple nematode *C. elegans* provides a convenient system in which to investigate host responses and defense against microsporidia infection. Interestingly, canonical *C. elegans* defense pathways, such as the conserved PMK-1 p38 MAPK pathway that provides defense against bacterial and fungal infections, are not important for defense against *N. parisii* [12], [14]. Thus, distinct immunity mechanisms may be involved in the *C. elegans* response to microsporidia. In addition to microsporidia, another natural intracellular infection has recently been described in *C. elegans*: wild-caught animals from Orsay, France, were shown to harbor a viral infection [15]. The Orsay virus is a positive strand RNA virus of the family *Nodaviridae*, and like *N. parisii* it appears to undergo its entire replicative cycle inside *C. elegans* intestinal cells. The RNAi pathway has been shown to provide defense against viral infections in *C. elegans* [15], [16], [17], [18], [19], but little else is known about host defense against this natural intracellular pathogen of *C. elegans*.

Defense against intracellular pathogens in diverse animal hosts is increasingly appreciated to involve ubiquitin-mediated degradation pathways [20], [21], [22], [23]. Ubiquitylation is the process by which an E3 ubiquitin ligase catalyzes the conjugation of a ubiquitin tag onto substrates, which can be further ubiquitylated to generate poly-ubiquitin chains [24]. Ubiquitylated substrates have a number of different fates, two of which involve degradation. The most well characterized fate is degradation by the proteasome, but larger substrates can be targeted for degradation by the process of autophagy, which is termed 'xenophagy' when it involves degradation of intracellular microbes [25], [26]. Recently, ubiquitin ligases that mediate ubiquitin targeting to human bacterial pathogens *Salmonella enterica* [21] and *Mycobacterium tuberculosis* [22] have been identified, and they, together with the autophagy pathway, are important for controlling levels of these intracellular pathogens [23], [27], [28], [29]. However, while several ubiquitin-mediated defense components and mechanisms have been defined, there are many unanswered questions about which host ubiquitin ligases are involved in targeting ubiquitin to different pathogens, how these systems are regulated, and their overall importance for defense *in vivo*.

One major class of E3 ubiquitin ligases includes the Skp1−Cul1−F-box protein (SCF) multi-subunit RING-finger type, which is a modular complex found throughout eukaryotes [30]. SCF ligases are usually composed of three core components (a cullin protein, Skp1, and a RING-containing subunit) and a variable F-box protein component, which enables recognition of different substrates depending on which F-box protein is associated with the complex [31]. Interestingly, the *C. elegans* genome has a greatly expanded and diversified family of F-box proteins (∼520 genes compared to 69 genes in humans), as well as other SCF components (21 Skp1-related genes compared to 1 in humans), suggesting they use SCF ligases to recognize an extremely diverse array of substrates [32], [33]. In particular, it has been proposed that *C. elegans* uses these SCF ligases to target toxins and intracellular pathogen proteins for degradation, and that the expanded *C. elegans* SCF ligase repertoire is the manifestation of a host/pathogen arms race between nematodes and their natural intracellular pathogens [32]. At the time this intriguing idea was proposed however, there were no known intracellular pathogens of *C. elegans* to test the role of ubiquitin-mediated responses in defense.

Here we describe the *C. elegans* host response to the natural intracellular pathogens *N. parisii* and the Orsay virus, and find a role for ubiquitin-mediated defense against both infections. We perform gene expression analyses of the transcriptional response to microsporidia infection and find that the response is strikingly similar to the response to viral infection, but not to extracellular pathogens. We see upregulation of SCF ligase components, which help to restrict microsporidia growth, and find that defense against microsporidia appears to rely on the proteasome, as well as the autophagy pathway. We find a subset of parasite cells targeted by host-derived ubiquitin, which relies partly on the SCF cullin component CUL-6. Notably, this ubiquitin targeting, as well as the role for ubiquitin-mediated defense, increases upon inhibition of microsporidia growth by anti-microsporidia drugs. These results suggest that *N. parisii* may suppress or evade ubiquitin-mediated host defenses. Interestingly, expression of specific infection-upregulated SCF ligase components is also upregulated by genetic or pharmacological inhibition of UPS function, suggesting that stress placed upon the UPS may be a hallmark of intracellular infection, and that hosts monitor UPS function to upregulate appropriate defenses during intracellular infection. Finally, we show that SCF ligase components, in particular CUL-6, promote defense against viral infection in *C. elegans*. Altogether, these studies show the involvement of ubiquitin-mediated defense and xenophagy against natural intracellular pathogens in a whole animal host, and provide insight into their regulation in response to infection *in vivo*.

## Results

### *C. elegans* transcriptional response to *N. parisii* infection is distinct from response to extracellular infection, but similar to response to viral infection

**Figure 1:**
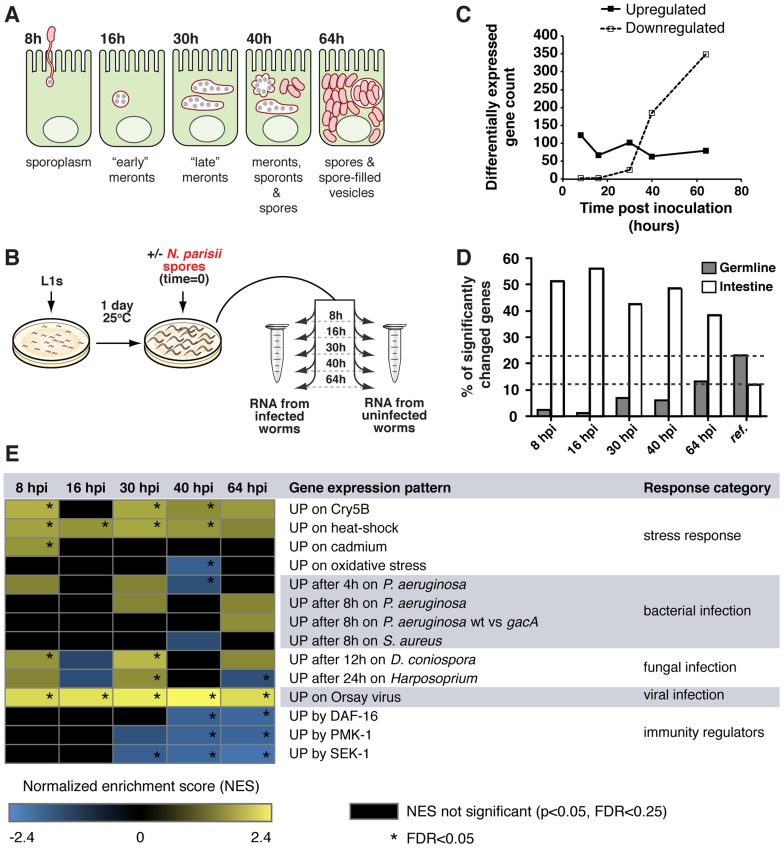
*C. elegans* gene expression during infection with *N. parisii*. A) Diagram of *N. parisii* infection stages in *C. elegans* intestinal cells. B) Synchronized populations of *fer-15;fem-1* sterile animals were inoculated with *N. parisii* spores and collected for RNA extraction at timepoints corresponding to specific stages of infection. Uninfected controls were included for each timepoint. C) Number of significantly (FDR<0.05) up- or downregulated *C. elegans* genes during infection with *N. parisii*. D) Proportion of intestine- and germline-associated *C. elegans* genes with significantly altered expression at each timepoint. The “reference” bars indicate intestine or germline associated genes as a percentage of the *C. elegans* genome (20,404 genes). At each timepoint, 39% to 56% of all highly regulated genes were associated with the intestine, which represents a significant enrichment (chi-squared test, p<1.03E-26, all comparisons), while germline genes were significantly underrepresented (chi-squared test, p<1.51E-05, all comparisons) (Figure 1D, Table S2). E) Correlations between genes regulated by *N. parisii* infection and genes upregulated by other pathogens, stressors and immunity pathways. Gene sets were compared using the GSEA software (see Table S5 for detailed summary of results) and normalized enrichment scores (NESs) with a relaxed significance threshold (FDR<0.25, p<0.05) are reported in the figure. A positive NES (yellow) indicates a correlation with genes upregulated in response to *N. parisii* infection, while a negative NES (blue) indicates a correlation with genes downregulated in response to *N. parisii* infection (see Materials and Methods for analysis details). Black indicates no significant (FDR<0.25, p<0.05) correlation, and an NES with FDR<0.05 is indicated with an asterisk.

We examined the *C. elegans* transcriptional response over the course of an infection with *N. parisii* using strand-specific deep sequencing of RNA (RNA-seq). Like other microsporidia, the life cycle of *N. parisii* is complex and its growth and replication takes place entirely inside the host cell (Figure 1A). Microsporidian spores initiate an intracellular infection by firing an infection apparatus called a polar tube, which pierces the host cell membrane and then injects into the host cell a nucleus and sporoplasm, which replicates as a stage called a meront. In the case of *N. parisii*, meronts become very large, multi-nucleate cells that replicate in direct contact with the cytoplasm. Meronts will eventually differentiate into spores and these spores then exit from infected cells to infect new hosts. We collected and sequenced cDNA from age-matched uninfected controls and infected animals at 8, 16, 30, 40 and 64 hours post inoculation (hpi) (Figure 1A, B), which are timepoints that correspond to specific stages of *N. parisii* infection as described in our previous study [34] (Table S1). A large number of *C. elegans* genes had significantly altered expression during *N. parisii* infection (edgeR, FDR<0.05, Table S2). The overall number of upregulated genes was relatively stable throughout infection, while the number of downregulated genes increased markedly with time (Figure 1C). To validate our RNA-seq studies, we also performed Affymetrix microarrays, which had substantial agreement in the genes found to be regulated by *N. parisii* infection (see Supplemental Text S1, Table S3). Notably, we found that a significant number of genes upregulated by infection were associated with the intestine, which is the site of *N. parisii* infection (Figure 1D).

Next, we compared genes regulated by *N. parisii* (Table S4) to gene sets regulated by infection with other pathogenic microbes, by treatment with non-biotic stressors, and by known immunity and stress-response pathways in *C. elegan*s [17], [35], [36], [37], [38], [39], [40], [41] (Figure 1E, Table S5, Table S6). Here, we used a well-established analytical method called Gene Set Enrichment Analysis (GSEA), which analyzes gene expression data at the level of gene sets instead of individual genes (see Materials and Methods) [42]. We found limited but significant correlations with gene sets upregulated by heat shock treatments, the pore-forming toxin Crystal protein-5B (Cry5B), and *Drechmeria coniospora* fungal infection, predominantly at the 30 hpi timepoint (Figure 1E). The heat shock pathway has been shown to play a role in resistance to bacterial pathogens as well as other stresses [43], [44]. However, despite the overlap between genes induced by heat shock and microsporidia, we found that *N. parisii* infection upregulated only two canonical heat shock protein-encoding genes, *hsp-17* at 30 hpi and *hsp-16.1/hsp-16.11* (which have identical sequence and are indistinguishable in RNA-seq data) at 64 hpi (Table S7, Figure S1A). Notably, there was almost no correlation between *C. elegans* genes upregulated in response to *N. parisii* infection compared to infections with the extracellular bacterial pathogens *Pseudomonas aeruginosa* and *Staphylococcus aureus*, the fungal pathogen *Harposporium*, or to genes affected by known *C. elegans* immunity regulators (Figure 1E). However, there was extensive correlation between genes downregulated by *N. parisii* and genes downregulated by other pathogens - for further discussion of this correlation, and other comparisons to previously published gene expression analyses see Supplemental Text S1 and Figure S2. Strikingly, we found a very strong correlation between genes most strongly upregulated by *N. parisii*, (e.g. genes of unknown function *C17H1.6* and *F26F2.1*) and genes upregulated by viral infection (Figure 1E, Figure S1B). Thus, *N. parisii* induces robust gene expression changes that are largely distinct from changes induced by extracellular pathogens, but share similarity to changes induced by the Orsay virus, which is another natural intracellular pathogen of *C. elegans*.

### *N. parisii* and viral infection upregulates expression of genes involved in ubiquitylation

To understand the nature of the *C. elegans* response to microsporidia infection, we analyzed the enrichment of gene ontology (GO) and Kyoto Encyclopedia of Genes and Genomes (KEGG) terms for the significantly induced and repressed genes [45], [46] (Table 1 and S8). Early during infection upregulated genes were enriched for GO terms associated with regulation of growth, while at later timepoints they were enriched for GO terms associated with the nucleosome, defense response, and structural components. At 30 hpi, upregulated genes were enriched for association with the ubiquitin-mediated proteolysis KEGG pathway (Table 1). To extend our analysis, we also identified specific enrichment of Pfam protein domains among *N. parisii* regulated genes (Table 1 and S8). At early times following infection these included two *Caenorhabditis* domains of unknown function, DUF713 and DUF684. Notably, genes upregulated at 8, 16 and 30 hpi were also enriched for the F-box, FTH (fog-2-homology), and MATH (meprin and Traf homology) protein-protein interaction domains, which are domains associated with ubiquitin-mediated proteolysis. For more details on regulated proteins containing these domains, analysis of gene enrichment at later time points, and analysis of downregulated genes, see Supplemental Text S1.

**Table 1:** Gene Ontology (GO), Kyoto Encyclopedia of Genes and Genomes (KEGG), and Pfam domain (PF) enrichment analysis of *C. elegans* genes upreguated by *N. parisii* infection.

Gene groups*	GO term, KEGG process, or Pfam domain	P-value
**UP at 8 hpi ** ***(126/129)***	PF05218:DUF713	1.04E-15
	PF00917:MATH	1.24E-09
	PF01827:FTH	2.36E-04
	PF05075:DUF684	4.94E-02
**UP at 16 hpi ** ***(66/68)***	PF00917:MATH	8.61E-08
	PF05218:DUF713	2.59E-07
	GO:0045927∼positive regulation of growth	2.07E-02
	PF01344:Kelch_1	2.44E-02
**UP at 30 hpi ** ***(105/108)***	PF01827:FTH	4.36E-05
	GO:0040008∼regulation of growth	8.77E-05
	PF05218:DUF713	3.62E-04
	PF00646:F-box	1.10E-03
	PF00917:MATH	1.94E-03
	PF00059:Lectin_C	3.57E-02
	KEGG: cel04120:Ubiquitin mediated proteolysis	1.73E-02
**UP at 40 hpi ** ***(64/66)***	PF00125:Histone	4.10E-04
	GO:0045927∼positive regulation of growth	4.72E-04
	GO:0000786∼nucleosome	7.48E-04
**UP at 64 hpi ** ***(79/81)***	PF00755:Carn_acyltransf	3.22E-02
	PF00335:Tetraspanin family	3.51E-02
	GO:0006952∼defense response	2.49E-02
	GO:0031589∼cell-substrate adhesion	3.52E-02
	GO:0045111∼intermediate filament cytoskeleton	3.73E-02

GO term, KEGG pathway, and Pfam domain enrichment was analyzed using online DAVID Bioinformatics Resources 6.7. To eliminate redundancy, each term had at least 30% of associated genes not associated with any other term with a more significant P-value. *For each gene group the number of genes included in the analysis out of the total differentially expressed genes is indicated.

Previously it had been hypothesized that F-box and MATH domain-containing proteins could function in *C. elegans* to target foreign pathogen proteins for proteasomal degradation, as part of SCF multi-subunit E3 ubiquitin ligases [32]. Indeed, we found that *C. elegans* SCF ligase components, Skp1-related (*skr*) genes *skr-4* and *skr-5*, were significantly upregulated at 30 hpi with *N. parisii* (Table S2), while *skr-3* and the cullin gene *cul-6* were also upregulated at 30 hpi over 6.5- and 5.5-fold respectively, although the difference was not significant (Table S4). While these SCF ligase components were not reported to be significantly upregulated in a published dataset of the wild-type *C. elegans* response to viral infection [17], we found that in the virus-susceptible *rde-1* strain of *C. elegans*, the SCF ligase components *cul-6*, *skr-3, skr-4, and skr-5* were upregulated in response to viral infection (data not shown). Overall, this increased expression of genes encoding SCF ligase components (see Table S9 for list of significantly upregulated ubiquitylation-associated genes) is consistent with ubiquitylation being upregulated in virus and microsporidia-infected animals.

### *N. parisii* growth is limited by SCF ligase components, the proteasome and autophagy

**Figure 2:**
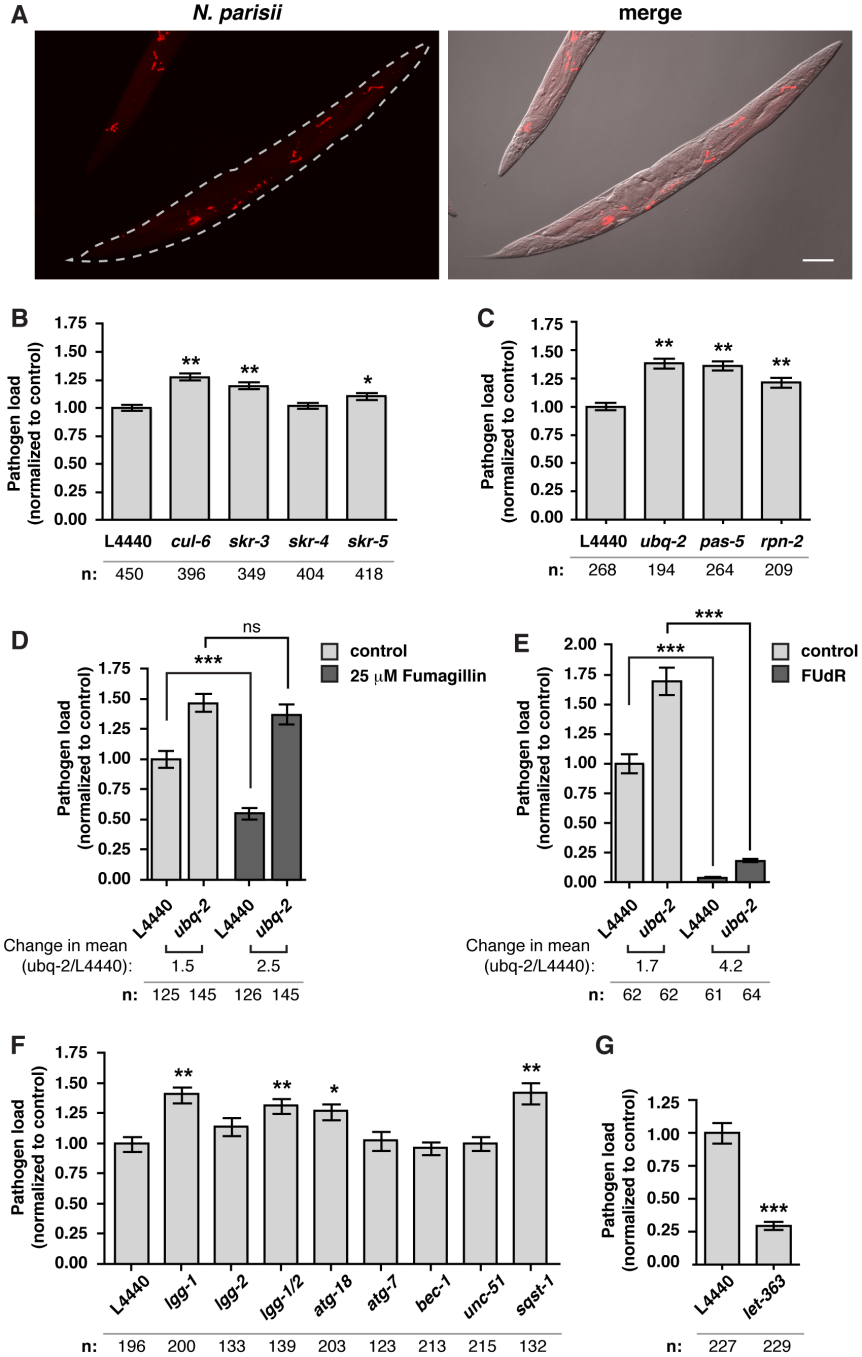
The SCF ligases, UPS and autophagy limit the growth of *N. parisii* in the *C. elegans* intestine. A) Fluorescence and bright field images demonstrating FISH staining with a probe against *N. parisii* rRNA used to quantify pathogen load in the *C. elegans* intestine following 24 hours of infection with *N. parisii*. Scale bar  = 100 µm. B–F) Quantification of pathogen load (see Materials and Methods) in nematodes treated with RNAi against SCF ligase components (B), against ubiquitin (*ubq-2*) and two components of the proteasome (*pas-5* and *rpn-2*) (C), against *ubq-2*, +/− fumagillin (D), against *ubq-2*, +/− FUdR (E), against autophagy components (F), and the *C. elegans* TOR ortholog *let-363* (G). Pathogen area occupying each RNAi-treated animal was normalized to mean L4440 control values. The number of animals analyzed for each condition (n) is indicated. Mean +/− SEM is shown for all analyzed animals (data for B, C, F are from three independent experiments, data for D, G are from two independent experiments and data for E are from one experiment). Each independent experiment was comprised of two separate populations of animals. Statistical significance was assessed using a one-way ANOVA with Dunnett's Multiple Comparisons Test for B, C, and F, with Bonferroni Multiple Comparison Test for D and E, and with student's t-test for G (***p<0.001, **p<0.01, *p<0.05).

To examine a functional role for genes induced by *N. parisii* infection we used RNAi to knock-down expression of specific genes, then infected these animals with *N. parisii* and measured pathogen load at 24 hpi by quantifying *N. parisii* rRNA FISH signal (Figure 2A, S3). We tested several genes highly induced by infection, as well as genes that belong to gene classes identified through our GO term and Pfam domain analysis. Knock-down of most genes showed little to no effect on pathogen load (see Supplemental Text S1 and Figure S4). When we examined whether the upregulated SCF ligase components have a functional role in defense against *N. parisii* infection we found a more substantial role. Because there are a large number of F-box proteins in the *C. elegans* genome (∼520 proteins), we focused on the core SCF ligase components that belong to smaller families, namely the Skp1-related *skr* family (21 proteins) and the cullin family (6 proteins), which likely have less functional redundancy than F-box proteins. In particular, we knocked down expression of *cul-6*, *skr-3, skr-4* and *skr-5* because these were upregulated upon *N. parisii* infection. Here, we found a modest but significant increase in pathogen load in *cul-6*, *skr-3* and *skr-5* RNAi-treated animals (Figure 2B), suggesting that these SCF ligase components limit the growth of *N. parisii* during infection.

After substrates have been ubiquitylated by ubiquitin ligases, they can either be degraded by the proteasome or by the autophagy pathway. First, we examined whether components of the proteasome may be acting downstream of SCF ligase components in limiting growth of *N. parisii*. We reduced expression of ubiquitin itself with RNAi against *ubq-2*, as well as two components of the proteasome: *pas-5* and *rpn-2*. In order for animals to develop properly, we introduced the RNAi in a diluted form at a late larval stage, infected animals and measured pathogen load. We found that reducing expression of any of these three genes led to an increase in pathogen load, suggesting that the UPS is important for defense against *N. parisii* (Figure 2C).

Because the effect of ubiquitin knock-down on pathogen load was modest, we hypothesized that, like other intracellular pathogens, *N. parisii* may suppress this defense system or subvert some aspects of the UPS to promote its replication. To test this hypothesis, we treated animals with drugs that block *N. parisii* growth but have minimal effects on adult *C. elegans* (see Supplementary Text S1) [47], [48]. First, we treated animals with a low dose of the anti-microsporidia drug fumagillin [49], [50], [51], [52], which limits *N. parisii* growth (Figure 2D and data not shown). After fumagillin treatment we found that *ubq-2* RNAi had a more robust effect on pathogen load (150% increase) than in the absence of this drug (50% increase) (Figure 2D). Similarly, *ubq-2* RNAi had a stronger effect on pathogen load when *N. parisii* growth was repressed with a DNA synthesis inhibitor, FUdR, (320% increase) than in the absence of this drug (70% increase) (Figure 2E). Taken together, these results suggest that the host UPS plays a greater role in controlling infection when pathogen growth is inhibited.

**Figure 3:**
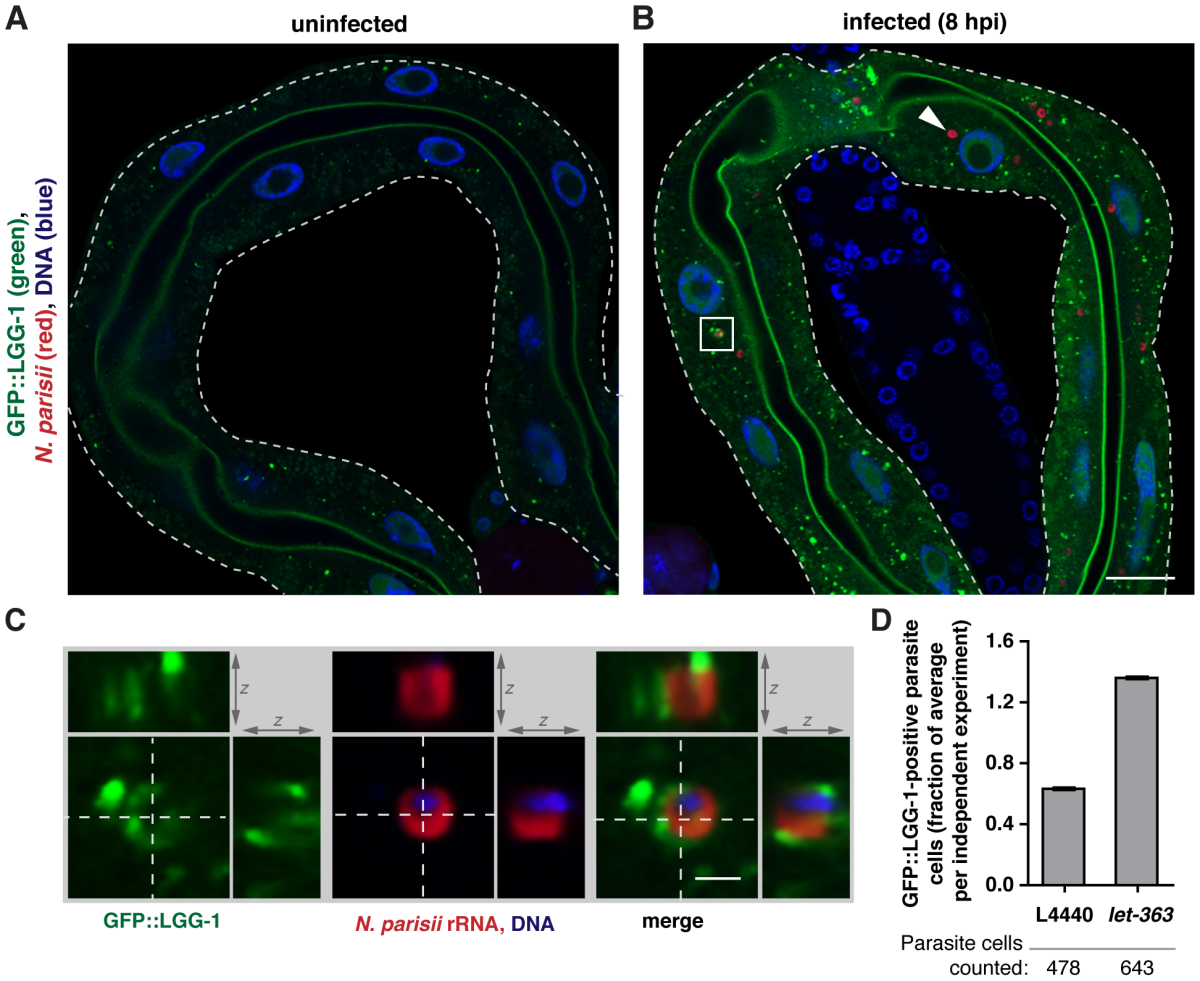
Targeting of *N. parisii* cells by the autophagy marker GFP::LGG-1 increases upon *let-363*/TOR RNAi. GFP::LGG-1-expressing transgenic animals were fixed and stained with a FISH probe against *N. parisii* rRNA (red) and DAPI for DNA (blue). A) Intestine of an uninfected nematode, and B) an *N. parisii*-infected nematode, 8 hpi, are shown. *N. parisii* parasite cells not colocalizing with GFP::LGG-1 (arrowhead) and surrounded by GFP::LGG-1 (boxed in area) are indicated. Scale bars  = 20 µm. C) Enlarged view of boxed in area from panel B, showing cross-section from two other dimensions. Scale bar  = 2 µm. D) Quantification of parasite cell colocalization at 8 hpi with wild-type GFP::LGG-1 following knockdown of *let-363* compared to L4440 vector control. Data are normalized for the average level of targeting in each independent experiment. Mean +/− SEM of two independent experiments is shown. Number of individual parasite cells assessed for colocalization is indicated.

We next investigated a role for the autophagy pathway in response to *N. parisii* infection. We used RNAi to knock-down expression of different autophagy components, infected these animals with *N. parisii*, and quantified pathogen load. Similar to the effects of knocking down components of the UPS, we found a modest but significant increase in pathogen load when expression of several key autophagy components was reduced (Figure 2F). Furthermore, RNAi of the *C. elegans* nutrient sensor TOR (Target Of Rapamycin) ortholog *let-363*, which activates autophagy in *C. elegans* [53], caused a dramatic 70% decrease in pathogen load (Figure 2G). To determine whether autophagy machinery was directed toward *N. parisii* cells, we examined localization of GFP-tagged LGG-1 (homolog of Atg8/LC3 in yeast/mammals) [54], a protein whose distribution is often used to assess autophagy [55]. We found that early during infection only 7% of parasite cells (25/360 parasite cells, n = 6 animals) were targeted by GFP::LGG-1 (Figure 3A–C). When animals were treated with *let-363* RNAi, we found that there was a greater than 2-fold increase in parasite cells targeted by GFP::LGG-1 (Figure 3D), consistent with this treatment causing an upregulation of the autophagy machinery directed toward *N. parisii* cells. Thus, the autophagy machinery appears to be targeted to *N. parisii* cells, and promotes resistance against infection.

One potential caveat to the results described above is that specific RNAi treatments might affect the feeding rates of nematodes, which could then result in changes in pathogen load simply due to differences in the initial dose of *N. parisii* spores ingested by these animals. To address this concern, we measured the accumulation of fluorescent beads in the intestinal lumen of animals fed dsRNA against the genes described above (Figure S5A–C). Importantly, RNAi against *let-363*/TOR, which causes decreased pathogen load, did not cause a decrease in the accumulation of fluorescent beads. In addition, RNAi against most autophagy genes that caused increased pathogen load did not cause an increase in accumulation of fluorescent beads. Furthermore, UPS RNAi, which increases pathogen load, did not increase fluorescent bead accumulation, and to the contrary, knock-down of *ubq-2* or *pas-5* marginally inhibited accumulation. Finally, feeding rates as measured by pharyngeal pumping were not affected by RNAi treatments, with the exception of *ubq-2* RNAi, which caused a decrease in feeding (Figure S5D–F). For further details on these controls, see Supplemental Text S1. Altogether, our data support the model that defense against *N. parisii* infection involves ubiquitylation components, the proteasome, and the autophagy pathway, although microsporidia appears to partially evade or suppress this ubiquitin-mediated response.

### Host ubiquitin targets some *N. parisii* cells, but most parasite cells escape this targeting

**Figure 4:**
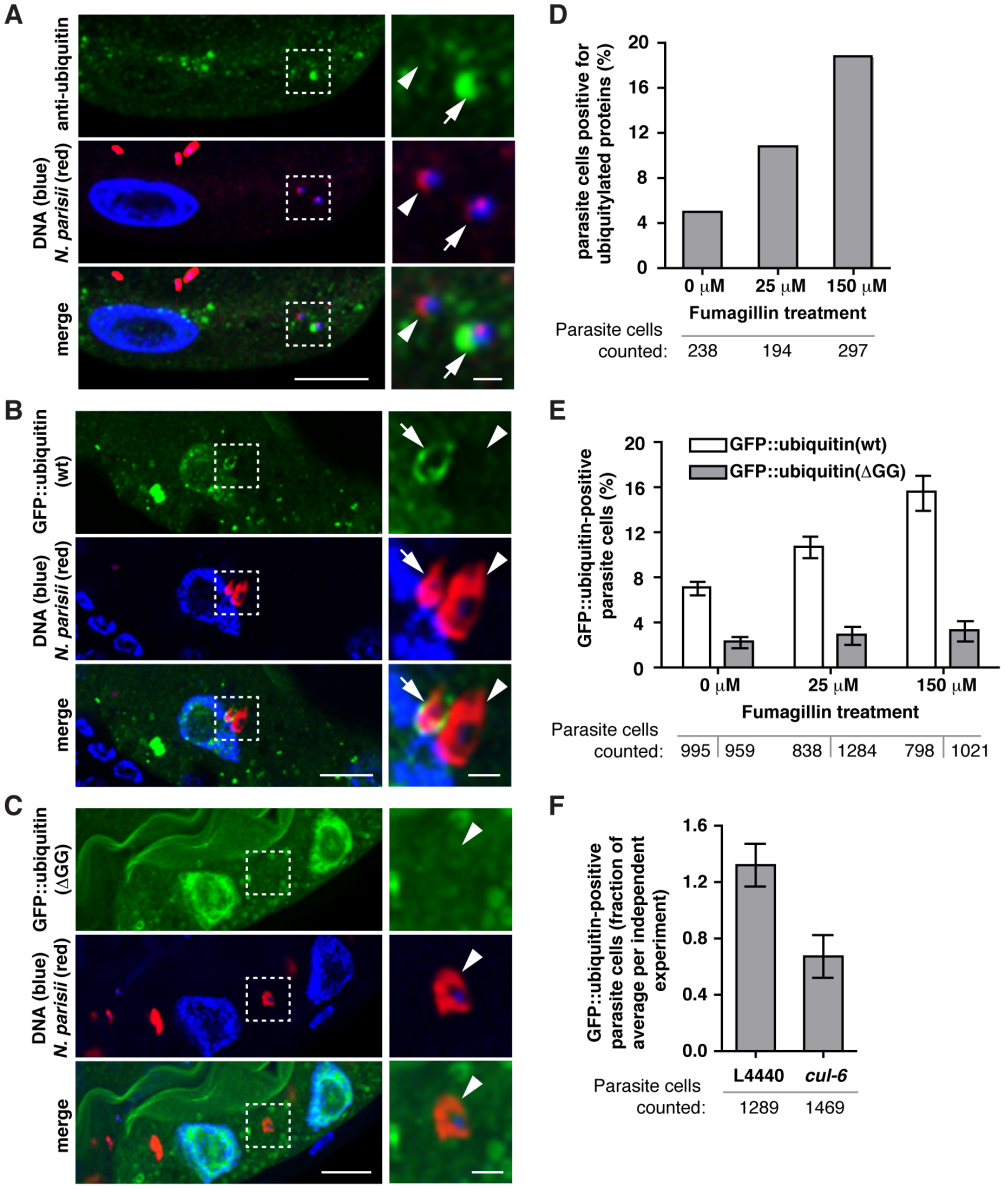
*N. parisii* cells are targeted by host ubiquitin early during infection. A–C) *C. elegans* intestines stained with a FISH probe against *N. parisii* rRNA (red), and DAPI for DNA (blue). A section of the image outlined by a dotted square is enlarged on the right and shows both an *N. parisii* parasite cell that colocalizes with ubiquitin (arrow), and one that does not (arrowhead). A) Animals were stained with an anti-conjugated-ubiquitin antibody, FK2 (green), and B, C) Transgenic *C. elegans* intestines expressing wild-type (B) or conjugation-defective mutant (C) GFP::ubiquitin (green). For A–C, scale bar  = 10 µm in main images and 2 µm in enlarged sections. D) Quantification of parasite cell colocalization at 12 hpi (see Materials and Methods for timepoint information) with FK2 antibody in the presence of increasing doses of fumagillin. Difference is significant: chi-squared test, p<6.3E-28, all comparisons. E) Quantification of parasite cell colocalization at 15 hpi with wild-type or mutant GFP::ubiquitin in the presence of increasing doses of fumagillin. Mean +/− SEM of two independent experiments is shown. F) Quantification of parasite cell colocalization at 15 hpi with wild-type GFP::ubiquitin following knockdown of *cul-6* RNAi compared to L4440 vector control. Targeting of ubiquitin to parasite cells was less robust and more variable in animals feeding on HT115 RNAi bacteria compared to OP50-1 *E. coli*, ranging from 10.6% to 2.2% in control animals, and 2.1% to 1.8% in *cul-6* RNAi treated animals and the data presented are normalized for the average level of targeting in each independent experiment. Mean +/− SEM of three independent experiments is shown. Number of individual parasite cells assessed for colocalization with ubiquitin is indicated.

To examine whether *N. parisii* itself is targeted by ubiquitin, we stained infected animals with the FK2 antibody, which recognizes ubiquitin that is conjugated to a substrate, and with a FISH probe against *N. parisii* rRNA to label the pathogen. Because *N. parisii* is a eukaryote, it contains its own ubiquitin, which is recognized by the FK2 antibody. However, distinct from this staining, we observed very strong accumulation of conjugated ubiquitin surrounding a subset of *N. parisii* meronts, with signal far above background of the microsporidia-derived ubiquitin (Figure 4A). To confirm that this ubiquitin was host-derived, we created a transgenic *C. elegans* strain that expresses a GFP::ubiquitin fusion protein under the control of an intestinal-specific promoter. Using these transgenic animals, we observed targeting of GFP::ubiquitin to parasite cells (Figure 4B). In contrast, we did not observe significant targeting to parasite cells by a conjugation defective GFP::ubiquitinΔGG fusion protein (Figure 4C, Figure S6). Altogether these experiments demonstrate that host ubiquitin is specifically targeted to *N. parisii* cells, where it is conjugated to a substrate.

The percentage of *N. parisii* cells specifically targeted by ubiquitin was relatively low: using the FK2 antibody we found only about 5% of pathogen cells were targeted by ubiquitin at 12 hpi (Figure 4D). Similarly, we found only about 7% of pathogen cells were targeted by GFP::ubiquitin (Figure 4E). Therefore, we examined whether *N. parisii* is suppressing or evading ubiquitin targeting by the host. If so, inhibiting the growth/vigor of the pathogen should cause an increased level of ubiquitin targeting. Indeed, we found increased targeting of ubiquitin to parasite cells after fumagillin treatment, with 16–18% of cells targeted (Figure 4D, E). This effect was dose-dependent, and was apparent both with the FK2 antibody, as well as the GFP::ubiquitin fusion protein. These results support the hypothesis that *N. parisii* is actively suppressing or evading ubiquitin targeting by *C. elegans,* and that after inhibition of *N. parisii* growth with an anti-microsporidia drug, the host is better able to target pathogen cells with ubiquitin.

Because the SCF ubiquitin ligase components *cul-6, skr-3* and *skr-5* serve to limit *N. parisii* growth (Figure 2B) we hypothesized that they could be responsible for ubiquitin targeting of parasite cells. Thus, we examined ubiquitin targeting to *N. parisii* cells in animals that had been treated with *cul-6* RNAi compared to the RNAi control (Figure 4F). Indeed, we found that *cul-6* RNAi had significantly reduced targeting of ubiquitin to *N. parisii* cells (two-tailed unpaired t-test, p<0.05). Thus, *cul-6* is important for efficient ubiquitylation of parasite-associated proteins, suggesting that *cul-6*-containing SCF ligases may mediate recognition of *N. parisii* infection by the host.

**Figure 5:**
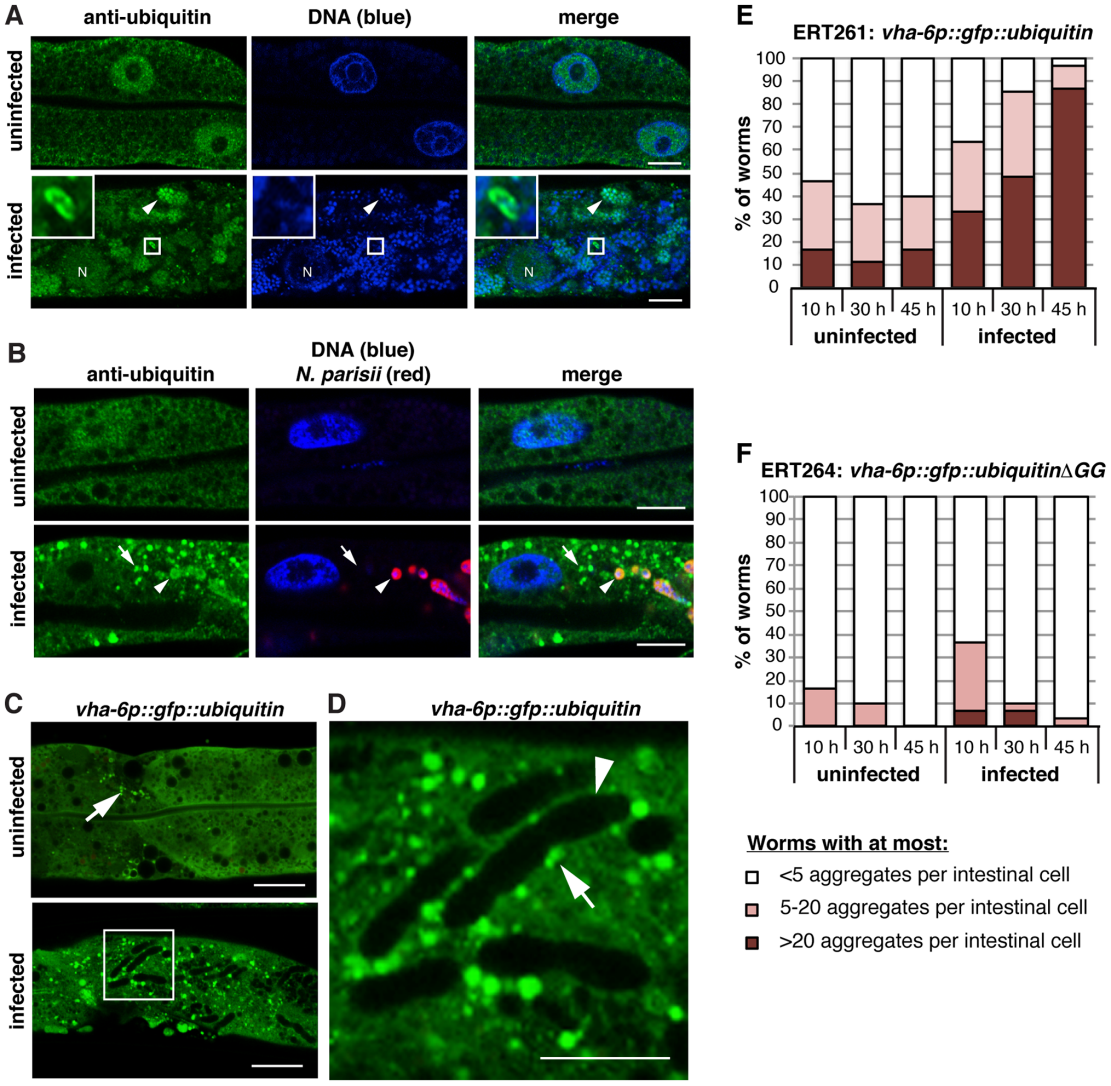
*N. parisii* cells are almost never targeted by ubiquitin later during infection, but ubiquitin forms clusters that accumulate in the *C. elegans* intestine. A,B) *C. elegans* intestines stained an anti-conjugated-ubiquitin antibody FK2 (green), and DAPI for DNA (blue): Panel B also includes a FISH probe against *N. parisii* rRNA (red). A) Sections of uninfected and *N. parisii*-infected *C. elegans* intestines. In the infected intestine an *N. parisii* spore labeled with the anti-conjugated-ubiquitin antibody is enlarged in box inset in upper left, and meront (arrowhead), and host nucleus (N) are indicated. Scale bar  = 10 µm. B) Sections of uninfected and *N. parisii*-infected *C. elegans* intestines (30 hpi) shown with ubiquitin cluster (arrow) and ubiquitin staining within an *N. parisii* meront (arrowhead) indicated. Scale bar  = 10 µm. C) Intestines of uninfected and infected animals expressing an intestinal GFP::ubiquitin transgene (48 hpi, grown at 20°C to prevent construct aggregation) are shown. Small GFP::ubiquitin aggregates are sometimes observed in uninfected animals (arrow). Scale bar  = 20 µm. D) Enlarged portion of box in panel C. Oblong *N. parisii* meronts are visible through the absence of green (arrowhead) and ubiquitin clusters associating with the meronts (arrow) are indicated. Scale bar  = 10 µm. E) Animals expressing the intestinal GFP::ubiquitin construct were infected with *N. parisii* and, together with control uninfected animals, fixed at the indicated times. Fixed animals were stained with a FISH probe against *N. parisii* rRNA to mark the infection and their intestinal cells were inspected for visible GFP::ubiquitin aggregates (30 transgenic animals were inspected per timepoint and condition). F) Animals expressing the intestinal control GFP::ubiquitinΔGG construct were treated and analyzed as in E.

The ubiquitin targeting of parasite cells described above was only observed at early timepoints of infection, when pathogen cells were small and mono-nucleate. When the pathogen cells grew bigger and became multi-nucleate meronts, we observed virtually no parasite cells targeted by ubiquitin or by autophagy (data not shown). Similarly, once meronts have differentiated into spores at later stages of infection, we found exceedingly few spores targeted by ubiquitin (Figure 5A). Although there was virtually no specific ubiquitin targeting to the parasite at these later stages of infection, we did observe an increased number of clusters of ubiquitylated proteins (Figure 5B–F). These clusters were dispersed throughout the infected intestinal cells, but in some cases were closely associated with *N. parisii*, although not encircling the parasite cells (Figure 5D). In addition, we found that infection caused increased clustering of the autophagy marker GFP::LGG-1 in regions distinct from the pathogen cells (Figure S7A–C) and found that GFP::LGG-1 partially colocalized with ubiquitylated protein clusters (Figure S7E–F). In order to determine whether this is a specific response, we examined GFP::LGG-1 upon infection with the extracellular bacterial pathogen *P. aeruginosa*, and did not find a significant increase in clustering (Figure S7D). Thus, as infection proceeds, an increased amount of conjugated ubiquitin and GFP::LGG-1 clusters accumulate in the host cytosol, and these markers are almost never seen specifically surrounding the pathogen cells.

### Perturbation of the UPS induces expression of SCF ligase components and other infection response genes in the intestine

**Figure 6:**
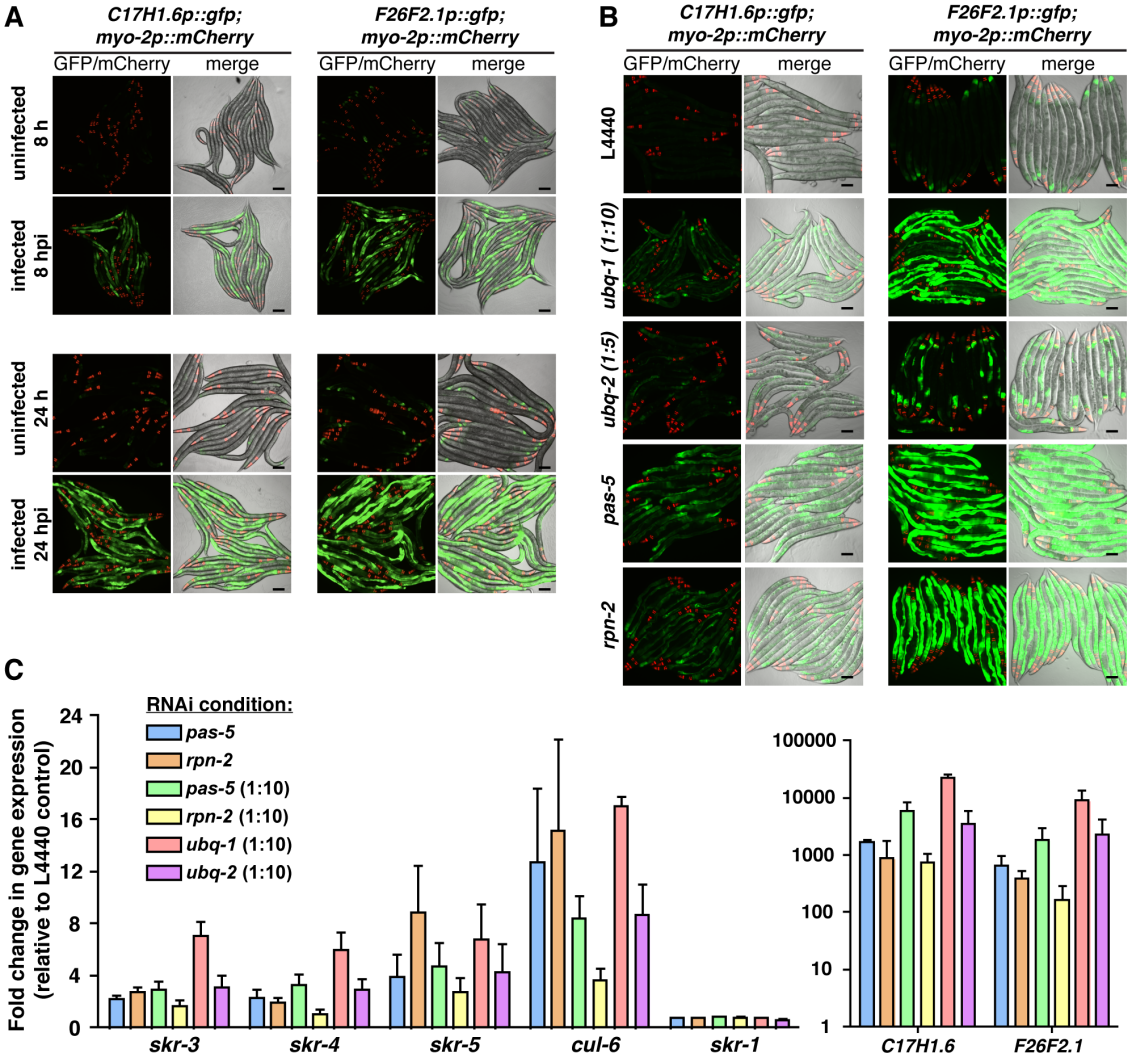
UPS perturbation induces similar gene expression responses to *N. parisii* infection. A) Expression of *C17H1.6p::gfp* and *F26F2.1p::gfp* in the intestine (pharyngeal *myo-2p::mCherry* expression is a marker for the presence of the transgene) following infection with *N. parisii* (8 and 24 hpi). Scale bars  = 100 µm. B) Expression of *C17H1.6p::gfp* and *F26F2.1p::gfp* following RNAi against *ubq-1, ubq-2*, *pas-5* and *rpn-2* in the absence of infection. C) Expression of endogenous mRNA of *C17H1.6* and *F26F2.1*, as well as the SCF ligase components *skr-1*, *skr-3*, *skr-4*, *skr-5* and *cul-6* following RNAi against *ubq-1, ubq-2*, *pas-5* and *rpn-2* in the absence of infection, as assessed by qRT-PCR. Due to the very large changes in expression of *C17H1.6* and *F26F2.1* genes, these are presented on a separate graph to allow for expansion of the y-axis and easier observation of expression changes in SCF ligase components. Mean +/- SEM of two to three independent experiments.

Recent studies have indicated that host cells monitor the functioning of core processes that are commonly perturbed by pathogen infection and that disruption of these processes can trigger defense-related gene expression by the host [56], [57], [58], [59], [60]. Because intracellular infection by *N. parisii* leads to an increase in ubiquitylated protein clusters, which may reflect an increase in demand on the UPS, we investigated whether perturbation of the UPS might be responsible for inducing gene expression changes upon *N. parisii* infection. To conveniently monitor gene expression *in vivo* and to examine where genes are induced upon *N. parisii* infection, we made promoter-GFP fusions for *C17H1.6* and *F26F2.1*, two genes of unknown function that are among the most highly upregulated genes at all infection timepoints (eg. at 8 hpi, *C17H1.6* and *F26F2.1* are upregulated 1.2×10^11^- and 1441-fold, respectively) (Table S2, S3). Expression of GFP driven by promoters of these genes was strongly induced in intestinal cells of infected animals by 8 hpi and even more robustly by 24 hpi (Figure 6A). These GFP reporters indicated that *N. parisii* infection drives expression of genes in intestinal cells of infected animals and provided convenient tools for monitoring expression of infection response genes.

To disrupt UPS function, we first performed RNAi knock-down of ubiquitin, *pas-5* and *rpn-2* in *C17H1.6p::gfp* and *F26F2.1p::gfp* transgenic animals. Strikingly, RNAi against the UPS components dramatically induced GFP expression in the intestine in both of these strains (Figure 6B). To confirm these results we performed qRT-PCR and saw levels of endogenous *C17H1.6* and *F26F2.1* mRNA transcripts also increased by UPS RNAi (Figure 6C). To perturb UPS function pharmacologically, we used the proteasome inhibitor MG-132 and similarly saw that this led to dramatic increase in *C17H1.6* and *F26F2.1* expression (Figure S8A–C). Because *C17H1.6* and *F26F2.1* are genes of unknown function, we extended these analyses to genes upregulated by intracellular infection that have predicted function, namely the genes that encode the SCF ubiquitin ligase components *skr-3, skr-4, skr-5* and *cul-6* (Figure 6C, Figure S8C). Similar to other infection response genes, we found that these genes were also induced by RNAi against the UPS, while another SCF component, *skr-1*, whose expression was not altered during microsporidia infection, was not affected (Figure 6C, Figure S8C). Thus, *C. elegans* appears to monitor efficacy of the UPS, and when this core process is disrupted it can trigger expression of a number of specific genes, including SCF components such as *cul-6* that are used by *C. elegans* to limit intracellular infection.

### SCF ligases promote anti-viral defense, although host UPS is required for viral replication

**Figure 7:**
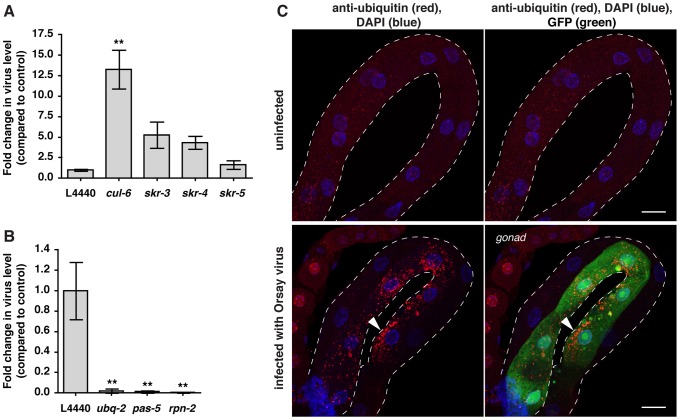
Ubiquitin-mediated host response and defense against Orsay viral infection in *C. elegans*. A) Viral pathogen load in nematodes treated with RNAi against the SCF ligase components *skr-3*, *skr-4, skr-5*, and *cul-6* compared to vector control RNAi (L4440), as assessed by qRT-PCR for viral transcript. Mean +/− SEM of three independent experiments shown. B) Viral pathogen load in nematodes treated with RNAi against *ubq-2*, *pas-5* and *rpn-2* analyzed as above. Mean +/− SEM of three independent experiments shown. C) Intestines of *F26F2.1p::gfp* transgenic animals were stained with the FK2 antibody against conjugated ubiquitin (red), and DAPI for DNA (blue). Intestines outlined with white dotted line. Animals infected with virus have increased ubiquitin clustering in some intestinal cells compared to uninfected animals (arrowhead), and also express the GFP reporter. Scale bar  = 20 µm. ** p<0.01.

The *C. elegans* gene expression response to *N. parisii* was most similar to its response to viral infection, including the upregulation of SCF ligase components (Figure 1E, Table S5, Table S6). Because of this similarity, we investigated whether the SCF ligases implicated in response to *N. parisii* also played a role in response to viral infection. Indeed, we found that *cul-6* RNAi caused a 13-fold increase in viral load, and *skr-3* and *skr-4* RNAi caused 5- and 4-fold increases in viral load respectively (Figure 7A), indicating that these SCF ligase components promote anti-viral defense. However, contrary to *N. parisii* infection, global inhibition of the UPS by RNAi-mediated knockdown of UPS components drastically reduced viral replication (Figure 7B). Many viruses exploit host UPS in order to replicate, for example to degrade host RNAi and immune signaling machinery, or to control function and stability of viral proteins [61], [62], [63], [64], [65], and thus the Orsay virus may likewise be hijacking this host pathway. Importantly, this result also suggests that the increased susceptibility to *N. parisii* infection of UPS-compromised nematodes (Figure 2C) is not likely just a result of general 'sickness' in these animals. Thus, the UPS appears to play two different roles in response to the Orsay virus, involving an unknown ligase(s) that promotes susceptibility to viral infection, and the *cul-6*, *skr-3* and *skr-4* SCF ubiquitin ligases promoting anti-viral defense.

Because *N. parisii* infection caused increased clustering of ubiquitylated proteins in *C. elegans* intestine, and robust gene expression changes in response to infection appeared to be a reflection of increased demand on the UPS, we investigated whether similar host responses occurred upon viral infection. Indeed, we found that infection with Orsay virus caused clustering of ubiquitylated proteins (Figure 7C), and clustering of GFP::LGG-1 (Figure S7A–D). Thus, infection with the Orsay virus induces similar cell biological changes as *N. parisii* infection. Furthermore, we found that viral infection induced the GFP reporter *F26F2.1p::gfp* (Figure 7C), which is also induced when the UPS is perturbed. Thus, it appears that the *C. elegans* transcriptional response to viral infection, like the response to *N. parisii* infection, involves surveillance pathways that detect perturbation of the UPS caused by infection, to upregulate defense gene expression.

## Discussion

### Ubiquitin-mediated defense against intracellular infection by *N. parisii* and the Orsay virus

**Figure 8:**
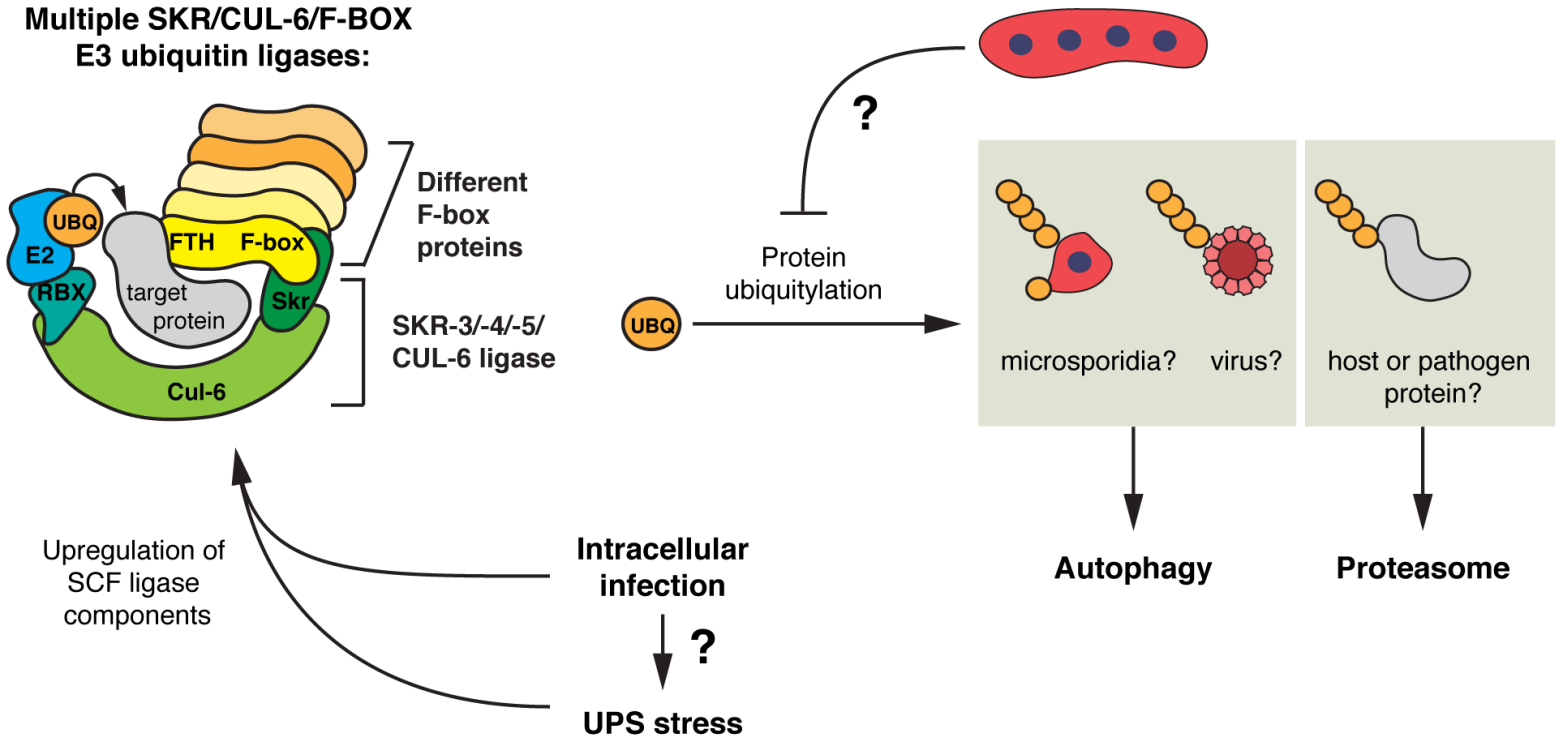
Model for SCF E3 ligases and ubiquitin-mediated responses to intracellular infection in *C. elegans*. Intracellular microsporidia or viral infection triggers the expression of SCF ligase components in *C. elegans*, including a large number of F-box genes, the cullin *cul-6*, and Skp1-related genes, *skr-3*, *-4*, and *-5*. Due to the modularity of the SCF ligase complex, many SCF ligases with vast substrate recognition potential may be formed, which could recognize pathogen-derived proteins or host proteins. Ubiquitylation of substrates leads to their degradation by the proteasome or by autophagy, with large substrates such as microsporidia cells, potentially viral particles, and protein aggregates (not shown), targeted by autophagy, and individual pathogen or host proteins by the proteasome. *N. parisii* parasite cells may be able to suppress or evade ubiquitylation. Both intracellular infection and UPS stress can induce SCF ligase components, and greater demand on the UPS during intracellular infection may contribute to upregulation of SCF ligase components. See Discussion for more details.

Based on our results we propose a model for the *C. elegans* intestinal response to intracellular infection (Figure 8), which highlights an important role for ubiquitin-mediated defense. In response to *N. parisii* infection, *C. elegans* upregulates expression of SCF ligase components, which restrict growth of the microsporidian pathogen *N. parisii*, as well as the Orsay virus. Restriction of *N. parisii* growth appears to also depend on the proteasome, as well as the autophagy pathway. While SCF ligase components such as CUL-6 have a substantial role in restricting growth of the virus, their more modest role in defense against *N. parisii* may be due to functional redundancy and/or the relatively inefficient targeting of ubiquitin to this pathogen. Inefficient targeting may be a result of suppression or evasion of host defenses by the parasite, as we find increased ubiquitin targeting of pathogen cells and a greater role for ubiquitin-mediated defense after treatment with drugs that inhibit *N. parisii* growth. Furthermore, we observe an increase in autophagy machinery targeting to *N. parisii* cells after activation of autophagy by inhibition of the TOR pathway. Interestingly, the increased demand on the UPS caused by intracellular pathogens like *N. parisii* and the Orsay virus may induce gene expression in response to infection, because genetic or pharmacological perturbation of the UPS upregulates expression of SCF ligase components and other genes that are induced by these intracellular infections.

SCF ligases comprise one of the major classes of E3 ubiquitin ligases that catalyze transfer of ubiquitin onto substrates. These ligases have very well characterized roles in controlling levels of endogenous proteins that regulate the cell cycle and development. Intriguingly, the expanded and diversified repertoire in *C. elegans* and plants of ubiquitin ligase adaptors such as F-box and BTB-MATH domain proteins, as well as other SCF components, has led to the hypothesis that these ligases may also be involved in recognition of foreign substrates. Our study with microsporidia and virus infection provides the first experimental support for this hypothesis. In particular, we see that the *C. elegans* SCF ligase components *cul-6* and *skr-3, skr-4*, and *skr-5*, mediate a defense response of *C. elegans* to *N. parisii* and virus infection. Previous reports indicated that CUL-6 and SKR-3 interact physically in a yeast two-hybrid assay, indicating these components could assemble *in vivo* to produce a functioning SCF ligase [33]. Moreover, we see targeting of ubiquitin to *N. parisii* cells that depends on the *cul-6* cullin component of the SCF ligase, which could conjugate host ubiquitin onto pathogen proteins or to host proteins that are associated with the pathogen cell. SCF ligases may also be involved in processing of proteins distinct from the pathogen cells, such as virulence factors that are secreted out of the pathogen cell into the cytosol. Another intriguing possibility is that SCF ligases are important for degrading inhibitory host proteins to trigger host innate immunity, analogous to ubiquitin-mediated degradation of IκB in NFκB signaling in mammals. However, the actual signaling proteins in *C. elegans* would be different because the NFκB transcription factor has been lost in this lineage [66]. Processing of host signaling proteins could occur in the clusters of conjugated ubiquitin we see later during infection, which are not associated with pathogen cells. Indeed, all of these possibilities are not mutually exclusive, and there are likely many roles for the SCF ligases and ubiquitin-mediated responses to intracellular infection in *C. elegans*.

### Perturbation of UPS function triggers upregulation of SCF ligases and other intracellular infection response genes

Our analysis of the gene expression response to *N. parisii* infection indicated that *C. elegans* has a very distinct response to this pathogen compared to previously described extracellular pathogens. Responses to extracellular pathogens like *S. aureus* and *P. aeruginosa* are marked by upregulation of secreted anti-microbials and detoxifying enzymes [37], [41], [67], [68], [69], which did not comprise a substantial part of the gene sets upregulated by *N. parisii*. Instead we found enrichment for genes associated with ubiquitylation (Table 1, S9), and that the response to *N. parisii* shared greatest similarities with the response to Orsay virus infection. The commonality of transcriptional response to these two very distinct pathogens (*N. parisii* is a eukaryotic organism with 2661 genes and the Orsay virus has only 3 genes) is quite striking, and our data indicate that some genes induced by infection such as SCF ligases can also be induced by perturbation of UPS function. Indeed, inhibition of the proteasome has been shown to induce stress response genes in other *C. elegans* studies as well [58], [59]. These results fit with the growing theme that *C. elegans* epithelial defense relies on monitoring of core host processes as an important cue to indicate the presence of pathogen attack [56], [57], [70], [71]. Such surveillance pathways are increasingly appreciated in mammalian defense as well, and may constitute a major mode by which hosts discriminate pathogens from other microbes [72], [73]. It is possible that surveillance of UPS function is responsible for controlling the transcriptional response to intracellular infection, although it is possible that UPS perturbation and infection are distinct triggers that converge to upregulate the same response genes.

Intracellular infection as well as perturbation of the UPS would be expected to cause substantial stress on the protein homeostasis (proteostasis) network of intestinal cells [74], [75], [76]. Intracellular infection by both *N. parisii* and virus should introduce a suite of foreign proteins into the host cell, may also cause damage to host proteins, and lead to activation of inducible immune responses. Any and all of these physiological changes may cause stress on the protein degradation and/or chaperone/folding systems of the host. This stress could explain the partial overlap we saw between the transcriptional response to intracellular infection and prolonged heat shock, a condition known to disrupt cellular proteostasis, although we saw an upregulation of only two *hsp* chaperones in response to infection (Table S7). In particular, *hsp-16.1*, which was significantly upregulated at 64 hpi when animal intestines are filled with parasite spores and large vacuoles (Figure 1A), has been shown to act in the Golgi where it helps to maintain cellular Ca^2+^ balance and protects cells against necrotic cell death triggered by heat as well as insults unrelated to thermal stress [43]. Further comparison between the responses to UPS stress and intracellular infection will likely shed light on mechanisms of cytosolic quality control and how they regulate defense against intracellular infection.

### *N. parisii* may suppress and/or evade recognition by the host ubiquitylation machinery

While ubiquitin-mediated defense does play a role in limiting *N. parisii* growth, it appears to be only a minor one. There are several reasons that could account for this small effect. First, because UPS components are essential for animal development and overall health we relied on partial knockdown of UPS components to compromise UPS function. Second, in analyses of genes that are not essential, such as SCF ligase components, there may be redundancy in the proteins involved in defense. Third, we anticipate that like other intracellular pathogens [77], [78] (for example the Orsay virus in this study), *N. parisii* may subvert host ubiquitylation machinery to promote its own growth. In this case, compromised host UPS would negatively impact both the replication of *N. parisii* as well as the ability of *C. elegans* to clear infection, yielding a small net change in pathogen load. Lastly, it is possible that *N. parisii* suppresses or evades the host ubiquitin-mediated defense. Consistent with this idea, *C. elegans* is better able to target ubiquitin to pathogens and induce their degradation when *N. parisii* is treated with drugs that slow its growth. Additionally, if *N. parisii* were suppressing *C. elegans* ubiquitin-mediated defenses, then genetic inhibition of these processes in the context of infection would only have a minor effect on pathogen resistance, while genetic activation could have a greater effect. Indeed, we found that activating autophagy through RNAi against *let-363*/TOR led to improved targeting and clearance of *N. parisii* cells, with a greater effect on resistance than autophagy inhibition. However, it is important to note that *let-363*/TOR is upstream of several other processes, including protein synthesis [79], which may also account for the increased resistance of this strain.

Other pathogens have been shown to actively suppress ubiquitin-mediated defenses of other eukaryotic hosts [21], [78], [80], [81], [82]. For example, in human cells, the bacterial pathogen *Salmonella enterica* suppresses ubiquitin-mediated host defenses with the GogB effector, which inhibits a human SCF ligase by interacting with Skp1 and the human F-box only 22 (FBXO22) protein, an interaction that impedes NFκB signaling and limits inflammation in infected cells [83]. Similarly, *N. parisii* might deploy effectors that block ubiquitylation of meronts, which are in direct contact with the cell cytosol of *C. elegans* intestinal cells and should be accessible to host ubiquitylation machinery. *N. parisii* might also evade ubiquitylation by the host by masking or simply lacking host-recognizable cues present during other intracellular pathogen infections. In particular, because *N. parisii* is itself a eukaryote, it may possess fewer pathogen-associated molecular patterns (e.g. bacterial peptidoglycan or lipopolysaccharide), which can be used by eukaryotes to recognize pathogens.

Microsporidia are increasingly recognized as natural pathogens of nematodes [84], [85], and *Nematocida* strains in particular have been isolated from multiple wild-caught *Caenorhabditis* nematodes [12]. It will be interesting to examine the interaction between other *Nematocida* pathogens and *Caenorhabditis* hosts to determine whether ubiquitin-mediated defenses have a greater or lesser role in those encounters, as part of the ever-shifting landscape of the host/pathogen arms race. Because microsporidia are obligate intracellular pathogens (which by definition cannot grow outside of host cells), it is imperative that they evade or suppress host defense pathways such as ubiquitylation to propagate the species. Thus suppression or evasion of host defense, together with extremely rapid intracellular replication [34], may be at the heart of why the Microsporidia have grown to be such a large and successful phylum able to infect virtually all animal hosts.

## Materials and Methods

### *C. elegans* and *N. parisii* culture conditions

All *C. elegans* strains were maintained on nematode growth media (NGM) and fed with *E. coli* strain OP50-1, as described [86]. *N. parisii* spores were prepared as previously described [87]. Briefly, *N. parisii* was cultured by infecting large-scale cultures of *C. elegans*, followed by mechanical disruption of worms and then filtering to isolate spores away from worm debris. The temperature-sensitive sterile strain CF512 *fer-15(b26);fem-1(hc17)* was used for RNA-seq and other experiments to prevent internal hatching of progeny at later infection time points. This strain was maintained using standard laboratory techniques at the permissive temperature of 15°C and shifted to 25°C for pathogen infection experiments [39]. The DA2123 *adIs2122[lgg-1p::gfp::lgg-1]* strain was a kind gift from Dr. Malene Hansen [88], [89].

### Generation of transgenic *C. elegans* strains

Promoter-GFP fusions for the *N. parisii* induced genes *C17H1.6* and *F26F2.1* were made using overlap PCR. Briefly, genomic DNA upstream of the predicted start for these genes was amplified (1273 bp for *C17H1.6* and 796 bp for *F26F2.1*) with PCR and then fused in frame to GFP amplified from pPD95.75. These promoter-GFP fusions were co-injected with the *myo-2p::mCherry* marker that labels pharyngeal muscle. Several independent transgenic lines carrying extrachromosomal arrays for these fusions were isolated and these lines induced GFP upon infection with *N. parisii*. One line for each fusion was integrated using psoralen/UV-irradiation to generate the integrated transgenic strains ERT54 *jyIs8[C17H1.6p::gfp; myo-2p::mCherry] ×* and ERT72 *jyIs15[F26F2.1p::gfp; myo-2::mCherry]*.

A GFP-tagged ubiquitin construct pET341 was generated using three-part Gateway recombination by fusing the intestinal-specific *vha-6* promoter to GFP at the N-terminus of ubiquitin (amplified from the *C. elegans ubq-1* gene), with a *unc-54* 3'UTR, introduced into destination vector pCFJ150 that encodes for a wild-type copy of *C. briggsae unc-119* gene under the control of the *unc-119* promoter. This construct was injected into EG6699 *ttTi5605 II; unc-119(ed9) III* mutant animals and transgenic progeny were recovered, to generate a multi-copy array strain ERT261 *jyEx128[vha-6p::gfp::ubiquitin cb-unc-119(+)];ttTi5605 II; unc-119(ed9)*. Likewise, construct pET346 was generated, which contains a mutant version of ubiquitin without its last two C-terminal glycines. This construct was injected into EG6699 to generate multi-copy array strain ERT264 *jyEx131[vha-6p::gfp::ubiquitinΔGG cb-unc-119(+)]ttTi5605 II; unc-119(ed9)*.

### *C. elegans* infections, RNA isolation and RNA-seq library construction

*C. elegans* infections, RNA isolation, and library construction are previously described [34]. Briefly, synchronized *fer-15(b26);fem-1(hc17)* L1s were grown for 24 hours at 25°C on 10-cm NGM plates seeded with OP50-1 *E. coli* and then infected with *N. parisii* ERTm1 spores. Infected and control *C. elegans* were harvested at appropriate times and total RNA was extracted using TriReagent (Molecular Research Center, Inc.). RT-qPCR and the Bioanalyzer assessed quality of RNA samples. Strand-specific libraries were constructed using the dUTP second strand marking method [90], [91].

### RNA-seq analysis

Reads were aligned using Bowtie[92] and transcript abundance estimated using RSEM [93]. Differentially expressed transcripts were identified using the edgeR Bioconductor package (Empirical analysis of digital gene expression data in R, v 3.0.8) [94]. FDR [95] cutoff was set to <0.05, which yielded lists of genes with >4-fold difference in expression. *C. elegans* reads comprised the majority of the infected sample reads, ranging from over 99% early during infection (8 and 16 hpi) to 71.6% at 40 hpi (Table S1). The progressive reduction in the fraction of *C. elegans* reads corresponded to replication of microsporidia in the *C. elegans* intestine resulting in increased contribution of parasite RNA to total RNA of each infected sample [34]. The number of expressed *C. elegans* genes in all samples ranged from 55.4% (64 hpi) to 62.1% (16 hpi) of the total genome (Table S1). Despite the growing input of parasite RNA, global *C. elegans* gene expression remained comparable between infected samples and uninfected controls, with the greatest absolute difference (3.61%) in total number of expressed genes, which occurred at 64 hpi (infected vs uninfected control).

### Tissue enrichment analysis

Based on previous studies, genes were classified as either intestinal-associated (as determined by fluorescence-activated nuclei sorting) [96], germline-associated (as determined by SAGE) [97], or neither. Very few germline specific/enriched genes were among the differentially expressed genes (Table S2) and therefore we used all genes expressed in germ lines detected by SAGE as the germline-associated class. We then compared the number of differentially expressed genes from each category to the number expected from the classification using the chi-squared test.

### GSEA analysis

Gene Set Enrichment Analysis (GSEA) v2.0 [42] was used to compare gene sets from relevant *C. elegans* expression studies to our RNA-seq data. The RNA-seq expression dataset file used to generate ranked gene lists (from most upregulated to most downregulated) based on changes in expression between infected and uninfected conditions is summarized in Table S4 while the compiled gene sets used for analysis are described in detail in Table S5. Genes from other studies were converted where necessary to WBGeneIDs according to Wormbase version WS235. Five independent analyses were performed, one for each infection timepoint, with 1000 permutations for each analysis. Results for gene sets with FDR<0.25 and nominal p-value<0.05 were compiled into a graphical representation based on their NES-values, and for gene sets where the NES was not considered significant a value of zero was assigned (Table S6).

### *N. parisii* pathogen load measured by FISH

Experiments were performed at 25°C and for each condition two biological replicates were included. About 200 synchronized *fer-15(b26);fem-1(hc17)* L1s were grown on 6-cm plates for two days, feeding on a lawn of *E. coli* RNAi clones from the Ahringer library or the *skr-4* RNAi clone generated through amplification of *C. elegans skr-4* genomic sequence (using primers 5′ CCGAATTCGTCTCACGAAAAGTGATC - and 5′- CCGAATTCGGCGTTATACATTTATTCAA) and cloned into the L4440 RNAi vector using EcoRI restriction sites. Animals were then infected with 2 million spores, fixed in 4% paraformaldehyde (PFA) 24 hpi, and stained with MicroB FISH probe against *N. parisii* rRNA as previously described [12], [34]. Stained animals were mounted on glass slides in Vectashield with DAPI (Vector Laboratories) and imaged using a Zeiss AxioImager microscope with a 10× objective. Exposure times were kept the same for all samples within a single experiment. For all experiments except for ones in Figures 2B, 2G and S4, where a custom fully automatic method for estimating pathogen load written in Matlab was used (see Figure S2 and Supplemental Methods in Text S1), images were analyzed semi-manually using ImageJ software, where the nematode body area, and the area of pathogen contained within were determined using two different thresholds of the MicroB FISH signal (a relaxed threshold to recognize the background staining of the animal body, and a stringent threshold to specifically recognize the pathogen). Due to developmental defects caused by knockdown of UPS components, for experiments targeting the UPS, animals were first grown for one day on *E. coli* strain OP50-1, and then transferred to plates seeded with UPS RNAi clones diluted with the L4440 RNAi vector control (1∶10 for *ubq-2*, 1∶5 for *pas-5*, and 1∶20 for *rpn-2*). *C. elegans* has two genes encoding for ubiquitin, *ubq-1* and *ubq-2*. The *ubq-2* RNAi clone was chosen for majority of experiments because it had less pronounced developmental defects then animals fed with RNAi against *ubq-1* (data not shown). After one day on RNAi, animals were infected and processed as described above. For fumagillin and FUdR experiments, animals were grown, infected, and processed as described above, except at 8 hpi, 0 or 25 µM of fumagillin (Medivet Pharmaceuticals Ltd.) or 0 or 2.6 µg/µL of FUdR (Acros Organics) in 250 µL of M9 with 0.1% Triton-X was spread onto plates containing the animals for a final concentration of 0 to 0.26 µg/mL (fumagillin) and 0 to 59 µg/mL (FUdR) present for the remainder of the experiment (an additional 16 hours).

### Conjugated-ubiquitin immunofluorescence and quantification of colocalization with *N. parisii*

To quantify ubiquitin colocalization with microsporidia, about 200 synchronized *fer-15(b26);fem-1(hc17)* L1s were grown on 6-cm plates for 2 days at 25°C, and then were infected with 5 million *N. parisii* spores. At 8 hpi, the infected animals were treated with 250 µL of 0 µM, 25 µM, or 150 µM of fumagillin in M9 with 0.1% Triton-X (fumagillin final plate concentrations of 0 µg/mL, 0.26 µg/mL, or 1.56 µg/mL). At 12 hpi, animals were anesthetized with 10 mM levamisole, their intestines dissected out, and fixed for 15–30 min in 4% PFA. The intestines were stained with MicroB FISH probe against *N. parisii* rRNA, followed by staining with FK2 antibody (Millipore), and secondary antibody staining with FITC goat anti-mouse IgG (Jackson ImmunoResearch). Stained intestines were mounted in Vectashield with DAPI (Vector Laboratories) and imaged. For each condition, *z*-stacks spanning the width of twelve intestines were taken, and colocalization between each imaged parasite cell and the FK2 antibody was determined. All images, unless specified otherwise, were captured using a laser scanning confocal microscope with a 40× oil immersion objective (Zeiss LSM 700, equipped with an AxioCam digital camera and Zen 2010 acquisition software). Images were imported into Adobe Photoshop and assembled using Adobe Illustrator.

For ubiquitin immunofluorescence at different stages of infection, animals were infected with *N. parisii* as described for RNA-seq. After 30 or 40 hpi, animals were anesthetized with 10 mM levamisole, their intestines dissected out, and fixed for 30 min in 4% PFA. The intestines from the 30 hpi infected and uninfected control samples were stained as described above. Intestines from the 40 hpi infected and control samples were stained directly with antibodies without FISH staining. Stained intestines were mounted in Vectashield with DAPI (Vector Laboratories) and imaged.

### GFP::ubiquitin imaging and quantification of colocalization with *N. parisii*

To quantify GFP::ubiquitin colocalization with microsporidia, about 200 synchronized ERT261 or ERT264 L1s were grown on 6-cm plates, seeded either with OP50-1 *E. coli* or control L4440 and *cul-6* RNAi clone, for 36 hours at 20°C and then infected with 5 million *N. parisii* spores. At 10 hpi, the infected animals were treated with 250 µL of 0 µM, 25 µM, or 150 µM of fumagillin in M9 with 0.1% Triton-X, and at 15 hpi animals were fixed in 4% PFA, stained with MicroB FISH probe against *N. parisii* rRNA, mounted in Vectashield with DAPI, and imaged as described above. For each condition and experiment, *z*-stacks spanning the width of twenty to eleven ERT261 and seven to ten ERT264 intestines were taken, and colocalization between each imaged parasite cell and GFP was determined. For RNAi experiments, eight to ten ERT261 animals were imaged for each condition and experiment.

For imaging of GFP::ubiquitin in live animals, synchronized ERT261 animals expressing the intestinal GFP::ubiquitin construct were grown and infected at 20°C to minimize ubiquitin aggregate formation in uninfected controls. Synchronized animals were grown for 24 hours on 6-cm plates prior to inoculation with 2 million *N. parisii* spores and 48 hpi were mounted on agarose pads, anesthetized with 1 mM levamisole and imaged. For quantification of GFP::ubiquitin aggregates, synchronized ERT261 or ERT264 animals were grown at 20°C for 31 hours on 6-cm plates prior to inoculation with 1 million spores. At 10, 30 and 45 hpi, animals were fixed with PFA and stained with MicroB FISH probe as described above. Stained animals were mounted in Vectashield with DAPI and viewed directly with a laser scanning confocal microscope with a 40× oil immersion objective (Zeiss LSM 700).

### Imaging of promoter-GFP reporter strains

To image promoter-GFP reporter strains, synchronized ERT54 and ERT72 L1s were grown for 24 hours at 25°C and infected with 10 million *N. parisii* spores on 6-cm plates. Infected and control worms were anesthetized with 1 mM levamisole, mounted on agar pads, and imaged at 8 and 24 hpi using a Zeiss AxioImager microscope. For RNAi experiments, synchronized ERT54 and ERT72 L1s were grown for 48 hours at 20°C on plates seeded with RNAi clones and imaged as described above. For MG-132 experiments, synchronized ERT54 and ERT72 L1s were grown for 24 hours at 20°C, incubated on a nutator at room temperature for six hours in M9 with 0.1% Triton-X and 0 µM, 500 µM, or 1mM MG-132, and then imaged as described above.

### qRT-PCR

To measure endogenous mRNA expression changes due to UPS component knockdown, synchronized *fer-15(b26);fem-1(hc17)* L1s were grown at 20°C for 48 hours on RNAi bacteria, and then collected in TriReagent (Molecular Research Center, Inc.) for RNA extraction. To measure endogenous mRNA expression changes due to pharmacological proteasome inhibition, synchronized *fer-15(b26);fem-1(hc17)* L1s were grown 24 h at 20°C, incubated on a nutator at room temperature for six hours in M9 with 0.1% Triton-X and 0 µM or 500 µM MG-132, and then collected in TriReagent for RNA extraction. RNA extraction, reverse transcription, and qRT-PCR were performed as previously described [41]. qRT-PCR primer sequences are available upon request. Each biological replicate was measured in duplicate and normalized to the *snb-1* control gene, which did not change upon conditions tested. The Pffafl method was used for quantifying data [98].

### Orsay virus preparation, infections, measurements of viral load, and immunofluorescence

Virus stock for infections was prepared as described previously [5], with minor modifications. Briefly, the virus-susceptible *rde-1(ne219)* nematodes were grown in large-scale cultures until just starved, mechanically disrupted, and filtered through a 0.2 µm filter to separate the virus away from nematode debris. When spread on a 6-cm plate in a 250 µL volume, the 1∶50 dilution of this filtrate was the maximum dilution tested that turned on the *F26F2.1p::gfp* reporter in all animals 24 hpi at 25°C (data not shown). These conditions were used for all viral infections. To measure changes in viral load upon RNAi-mediated knockdown of *C. elegans* genes of interest, the viral RNA1 levels were measured using primers GW195 and GW194 [5] and compared to those found in L4440 controls. For these experiments, *fer-15(b26);fem-1(hc17)* animals were grown and treated with RNAi the same as for the *N. parisii* pathogen load experiments, except about 300 synchronized L1 animals were used per 6-cm plate and following 24 hours of infection with the virus, animals were collected for RNA extraction and qRT-PCR. Intestine dissections from ERT72 animals and immunofluorescence with FK2 anti-conjugated-ubiquitin antibody were performed as described above, except the secondary antibody used was the Cy3 goat anti-mouse IgG (Jackson ImmunoResearch).

### Accession numbers/gene names

*C. elegans* genes analyzed:

*cul-6, skr-3, skr-4, skr-5, ubq-1, ubq-2, pas-5, rpn-2, lgg-1, lgg-2, atg-18, sqst-1, let-363, C17H1.6, F26F2.1, skr-1, C17H1.14, F26F2.4, Y39G8B.5, sdz-6, T08E11.1, W08A12.4, ZC196.3, Y94H6A.2, his-10, his-16*

RNA-seq data are part of NCBI BioProject #PRJNA163569.

## Supporting Information

Figure S1 **Overlap between genes upregulated by** ***N. parisii*** **infection and other stressors.** A) Venn diagram showing an overlap of genes significantly upregulated during *N. parisii* infection at 8 hpi (blue), genes upregulated by two heat shock conditions (green) (a prolonged condition 1 [38] and an acute condition 2 [99]), and genes belonging to the Heat Shock Protein (*hsp*) gene class (pink). For gene names and complete analysis at all timepoints, see Table S7. B) Venn diagram showing an overlap of genes significantly upregulated during *N. parisii* infection at 8 hpi (blue), and genes upregulated by infection with the Orsay virus (yellow). C) Venn diagram showing an overlap of genes significantly upregulated during *N. parisii* infection at 8 hpi (blue), and genes upregulated by prolonged heat shock (green) or Cry5B (orange).
(TIF)

Figure S2 **Correlations between genes regulated by** ***N. parisii*** **infection and genes downregulated by other pathogens, stressors and immunity pathways.** Gene sets were compared using the GSEA software (see Table S5 for detailed summary of results) and normalized enrichment scores (NESs) with a relaxed significance threshold (FDR<0.25, p<0.05) are reported in the figure. A positive NES (yellow) indicates a correlation with genes upregulated in response to *N. parisii* infection, while a negative NES (blue) indicates a correlation with genes downregulated in response to *N. parisii* infection (see Materials and Methods for analysis details). Black indicates no significant (FDR<0.25, p<0.05) correlation, and an NES with FDR<0.05 is indicated with an asterisk.
(TIF)

Figure S3 **Comparison of ImageJ and Matlab methods of image analysis for pathogen load as measured by FISH.** For automated Matlab analysis nematodes were fixed, stained with a FISH probe against *N. parisii* rRNA (red), a FISH probe against *C. elegans* rRNA (green), and DAPI for DNA (blue), and imaged using a 2.5× objective. DAPI and the *C. elegans* rRNA FISH probe signals were used to recognize each animal and the signal from the *N. parisii* FISH probe was used to determine the area of each animal occupied by the pathogen. Animals were manually censored for proper analysis by the software. A–E) Example of a single image analyzed by the Matlab program, showing the *N. parisii* FISH signal (A), the *C. elegans* rRNA (green) and DAPI (blue) signals (B), a merged image of all fluorescence and bright-field channels (C), and animals selected for analysis by the program (pink) (D). Note that animals touching bubbles, each other, or crossing the edges of the image, are not selected. E) An example of the analysis of a single infected nematode from a boxed in area in the panels above. The raw *N. parisii* signal (left panel), and a joint image generated by the program with areas recognized as the animal body (cyan) and the parasite (orange) (right panel) is shown. F) Animals from the same slides from a single experiment (one of independent experiments presented in Figure 2B) were imaged with a 10× objective and their pathogen load was measured with ImageJ, or they were imaged with the 2.5× objective and analyzed with the Matlab program. Mean pathogen area occupying each animal, normalized to mean L4440 control values +/− SEM, is shown. The number of animals analyzed for each condition (n) is indicated. Statistical significance was assessed using a two-way ANOVA with a Bonferroni posttest. While RNAi treatment significantly affected the results (p<0.0001), for each RNAi condition the analysis method did not yield statistically significant differences, p>0.05 (ns).
(TIF)

Figure S4 **Analysis of pathogen load in animals treated with RNAi against genes upregulated during** ***N. parisii*** **infection.** Quantification of pathogen load (see Materials and Methods) in nematodes treated with RNAi against the indicated genes. A, B) Synchronized animals were grown for two days on dsRNA-expressing bacteria. (C) Synchronized animals were grown for one day on OP50-1 *E. coli* followed by one day on dsRNA-expressing bacteria. Pathogen area occupying each RNAi-treated animal was normalized to mean L4440 control values. The number of animals analyzed for each condition (n) is indicated. Mean +/− SEM is shown for all analyzed animals (data are from at least one independent experiment comprised of two separate populations of animals). Statistical significance was assessed using a one-way ANOVA with Dunnett's Multiple Comparisons Test (**p<0.01, *p<0.05).
(TIF)

Figure S5 **Analysis of animal feeding rates by quantification of fluorescent bead accumulation and pharyngeal pumping under different RNAi conditions.** A–C) RNAi-treated animals were fed fluorescent beads mixed with *N. parisii* spores for 30 min, fixed with PFA, and the fluorescence of accumulated beads in each animal was measured using the worm sorter. Red fluorescence was normalized to animal size and mean L4440 control values. The number of animals analyzed for each condition (n) is indicated. Mean +/− SEM is shown (data are from two independent experiments comprised of two separate populations of animals). D–E) Quantification of pharyngeal pumping rates of animals grown on indicated RNAi clones (n = 10). Synchronized animals were grown for one day on OP50-1 *E. coli* followed by one day on dsRNA-expressing bacteria prior to analysis (A, D). Synchronized animals were grown for two days on dsRNA-expressing bacteria prior to analysis (B, C, E, F). Statistical significance was assessed using a one-way ANOVA with Dunnett's Multiple Comparisons Test (**p<0.01, *p<0.05).
(TIF)

Figure S6 **Western blot of** ***C. elegans*** **lysates indicate that GFP::ubiquitin fusion protein is conjugated onto substrates, while GFP::ubiquitinΔGG fusion protein is not.** Lysates from equal numbers of N2 animals, and transgenic animals expressing wild-type GFP::ubiquitin, or conjugation-defective GFP::ubiquitinΔGG in their intestines were probed with anti-GFP antibody. The antibody recognized monomeric GFP::ubiquitin, free GFP, as well as GFP::ubiquitin conjugated to target proteins in the strain expressing wild-type GFP::ubiquitin (apparent as a high molecular weight smear) but not mutant GFP::ubiquitinΔGG. Proteins non-specifically recognized by the GFP antibody are seen in the N2 lysate. Anti-actin antibody was used as a loading control.
(TIF)

Figure S7 ***N. parisii*** **infection causes clustering of the GFP::LGG-1 autophagy marker in the** ***C. elegans*** **intestine.** (A–B) GFP::LGG-1-expressing transgenic animals were fixed and stained with a FISH probe against *N. parisii* rRNA (red) and DAPI for DNA (blue). A) Intestine of an uninfected nematode, and B) an *N. parisii*-infected nematode, 24 hpi, are shown. GFP::LGG-1 clusters are indicated with arrows. Scale bars  = 10 µm. C) Quantification of GFP::LGG-1 clusters (see Materials and Methods) in animals infected with different doses of *N. parisii* spores at three different timepoints. For each condition, mean values from ten to twelve animals +/− SEM are shown. D) Quantification of GFP::LGG-1 clusters in live animals infected with *Pseudomonas aeruginosa* strain PA14, or grown on *E. coli* strain OP50 at three different timepoints. For each condition, mean values from four to eight animals +/− SEM are shown. E,F) GFP::LGG-1-expressing (green) transgenic animals were fixed and stained with FISH probes against *N. parisii* rRNA (blue), an anti-conjugated-ubiquitin antibody FK2 (red), and DRAQ5 for DNA (blue). Uninfected intestine (E) and infected intestine, 8 hpi (F), are shown. Scale bars  = 10 µm. Conjugated-ubiquitin aggregates colocalizing with GFP::LGG-1 (arrow), or not colocalizing (arrowhead), and a *N. parisii* parasite cells (dashed arrow) are indicated.
(TIF)

Figure S8 **Pharmacological perturbation of the UPS induces infection response gene expression.** A, B) Treatment with the proteasome inhibitor MG-132 induces expression of *C17H1.6p::gfp* (A) and *F26F2.1p::gfp* (B) in the absence of infection. Scale bars  = 100 µm. C) MG-132 induces expression of endogenous mRNA transcripts for *C17H1.6, F26F2.1, skr-3, skr-4, skr-5*, *cul-6*, but not *skr-1*, as assessed by qRT-PCR. Mean +/− SEM of two independent experiments shown.
(TIF)

Figure S9 **Infection with Orsay virus induces GFP::LGG-1 clusters.** GFP::LGG-1-expressing (green) transgenic animals were fixed and stained with FISH probes against the Orsay virus RNA (red), an anti-conjugated-ubiquitin antibody FK2 (blue), and DAPI for DNA (blue). A) Uninfected intestine and B) Orsay virus-infected intestine, 24 hpi, are shown. Scale bars  = 10 µm. C and D) Enlarged view of boxed in area from panels A and B, respectively, with conjugated-ubiquitin aggregates colocalizing with GFP::LGG-1 (arrow), or not colocalizing (arrowhead). Scale bars  = 5 µm.
(TIF)

Table S1 **RNA-seq statistics.**
(XLSX)

Table S2 **Infection-regulated genes, as determined by edgeR.**
(XLSX)

Table S3 **Microarray results for** ***N. parisii*** **-infected wild-type (N2)** ***C. elegans*.**
(XLSX)

Table S4 **Normalized RNA-seq fpkm values for all genes and samples.**
(XLSX)

Table S5 **Gene sets used in GSEA analysis and their sources.**
(XLSX)

Table S6 **Summary of GSEA results for infected and control samples at each timepoint.**
(XLSX)

Table S7 **List of heat shock-upregulated genes that are also significantly upregulated during** ***N. parisii*** **infection.**
(XLSX)

Table S8 **GO, KEGG, and Pfam enrichment analysis of genes downregulated by** ***N. parisii*** **infection.**
(XLSX)

Table S9 **List of ubiquitylation-associated genes significantly upregulated during** ***N. parisii*** **infection.**
(XLSX)

Text S1 **Supplemental text file describing:** Microarray analysis of genes regulated by *N. parisii* infection; Comparisons between genes regulated by *N. parisii* and gene sets regulated by other pathogens and stressors; GO terms enriched at later timepoints of infection with *N. parisii*; Functional analysis of *N. parisii*-upregulated genes; Enrichment of F-box, FTH and MATH domains in genes regulated by *N. parisii* infection; The drugs fumagillin and FUdR limit microsporidia proliferation within *C. elegans*; Feeding controls for RNAi treatments that affect pathogen load; Supplemental Materials and Methods (Affymetrix microarray analysis; DAVID analysis; Automatic method for estimating pathogen load; Feeding assay; Pharyngeal pumping rates; GFP::LGG-1 imaging during *N. parisii* infection; Orsay virus, GFP::LGG-1, and conjugated-ubiquitin imaging; GFP::LGG-1 puncta quantification; Western blot analysis); and References.
(DOCX)
