# Correction: Ubiquitin-Mediated Response to Microsporidia and Virus Infection in *C. elegans*

**DOI:** 10.1371/journal.ppat.1004371

**Published:** 2014-08-15

**Authors:** The PLOS Pathogens Staff

The fourth author's name is spelled incorrectly. The correct name is: Tiffany L. Dunbar. The correct citation is: Bakowski MA, Desjardins CA, Smelkinson MG, Dunbar TL, Lopez-Moyado IF, et al. (2014) Ubiquitin-Mediated Response to Microsporidia and Virus Infection in *C. elegans*. PLoS Pathog 10(6): e1004200. doi:10.1371/journal.ppat.1004200
