# Supplementary figures and images for: Ubiquitin-Mediated Response to Microsporidia and Virus Infection in C. elegans
Source: PLoS Pathog. 2014 Jun 19;10(6):e1004200. doi: 10.1371/journal.ppat.1004200 (PMC4063957; doi:10.1371/journal.ppat.1004200)

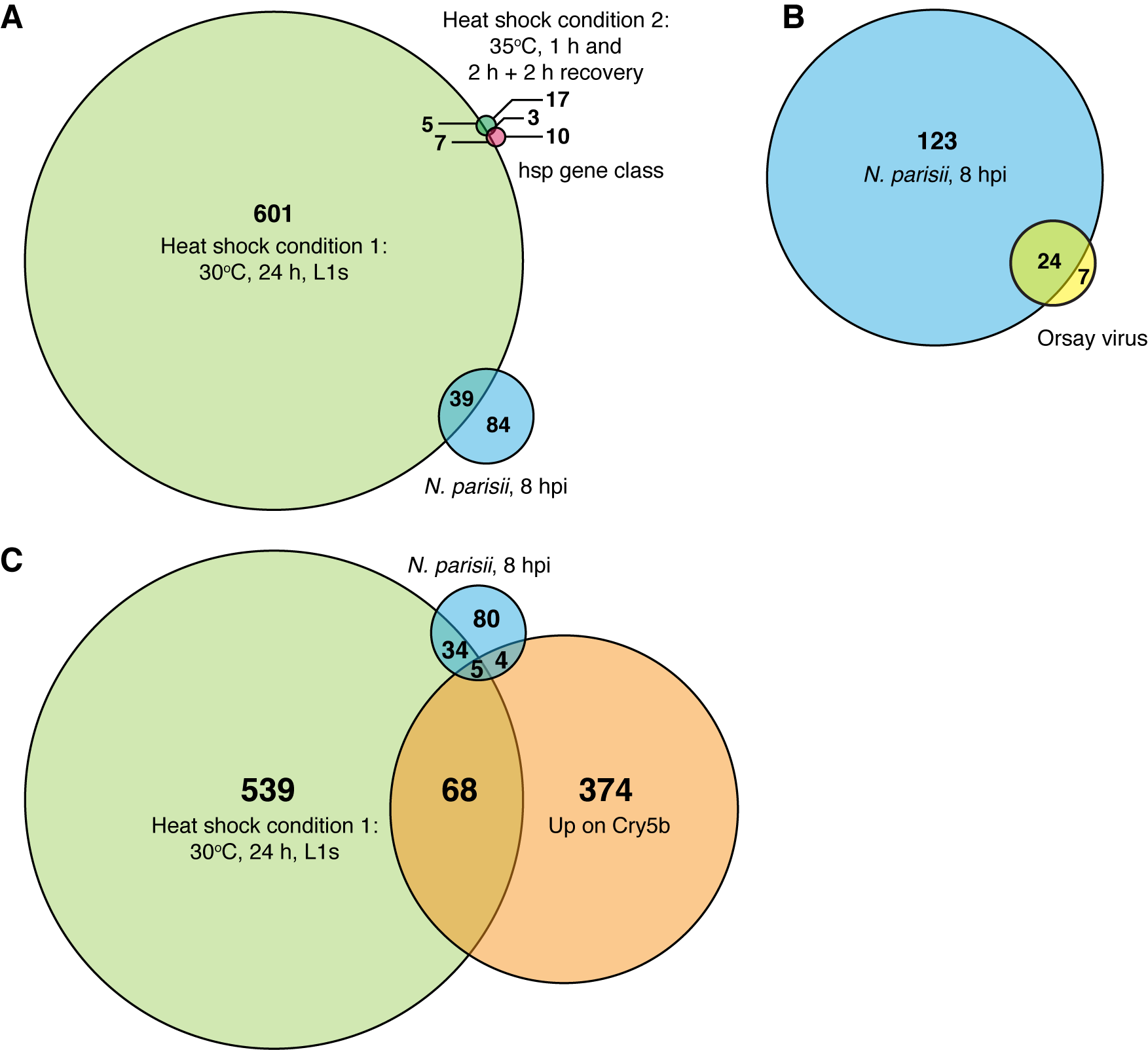

Supplement: Figure S1 — Overlap between genes upregulated by N. parisii infection and other stressors. A) Venn diagram showing an overlap of genes significantly upregulated during N. parisii infection at 8 hpi (blue), genes upregulated by two heat shock conditions (green) (a prolonged condition 1 [38] and an acute condition 2 [99]), and genes belonging to the Heat Shock Protein (hsp) gene class (pink). For gene names and complete analysis at all timepoints, see Table S7. B) Venn diagram showing an overlap of genes significantly upregulated during N. parisii infection at 8 hpi (blue), and genes upregulated by infection with the Orsay virus (yellow). C) Venn diagram showing an overlap of genes significantly upregulated during N. parisii infection at 8 hpi (blue), and genes upregulated by prolonged heat shock (green) or Cry5B (orange). (TIF) [file ppat.1004200.s001.tif]

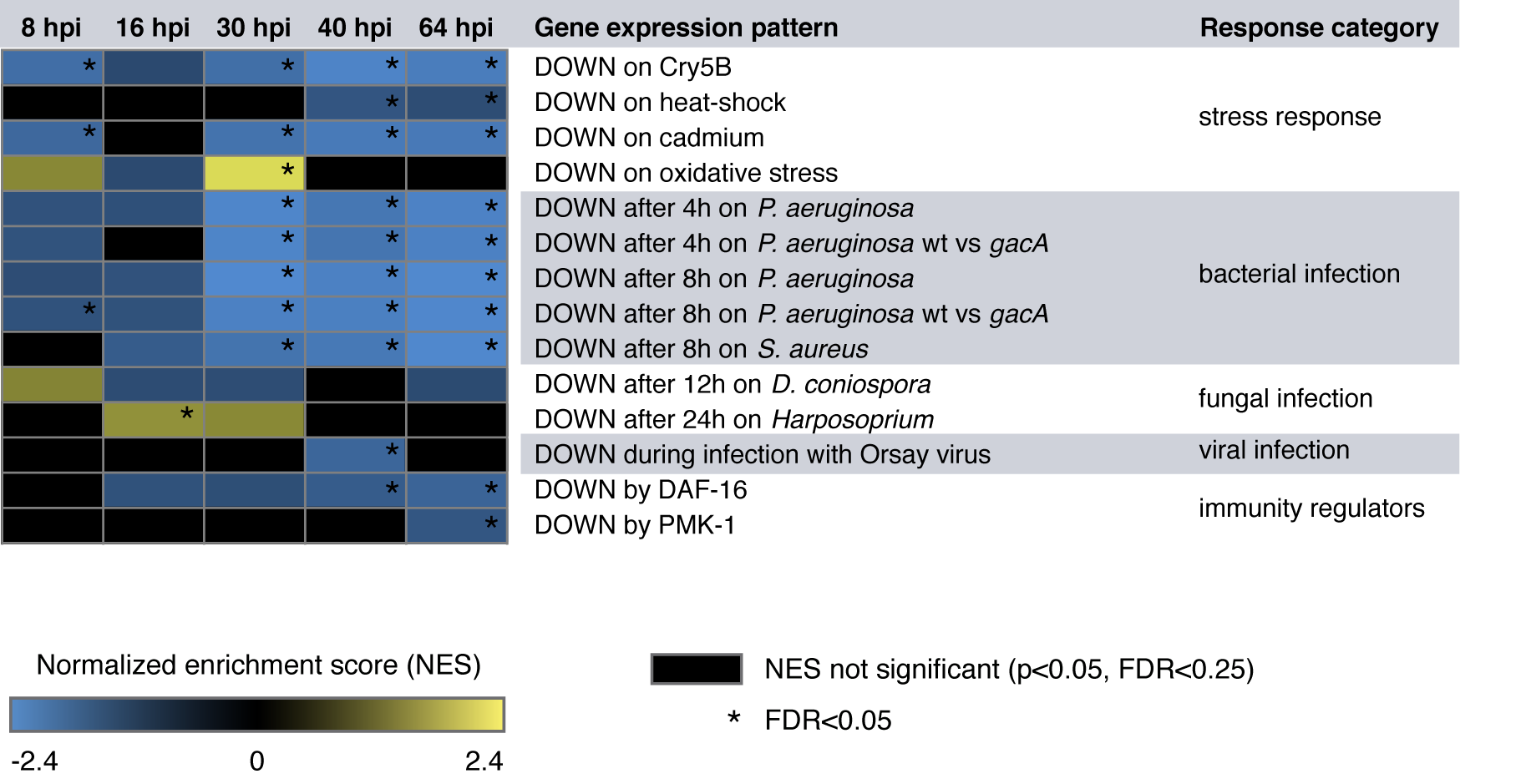

Supplement: Figure S2 — Correlations between genes regulated by N. parisii infection and genes downregulated by other pathogens, stressors and immunity pathways. Gene sets were compared using the GSEA software (see Table S5 for detailed summary of results) and normalized enrichment scores (NESs) with a relaxed significance threshold (FDR<0.25, p<0.05) are reported in the figure. A positive NES (yellow) indicates a correlation with genes upregulated in response to N. parisii infection, while a negative NES (blue) indicates a correlation with genes downregulated in response to N. parisii infection (see Materials and Methods for analysis details). Black indicates no significant (FDR<0.25, p<0.05) correlation, and an NES with FDR<0.05 is indicated with an asterisk. (TIF) [file ppat.1004200.s002.tif]

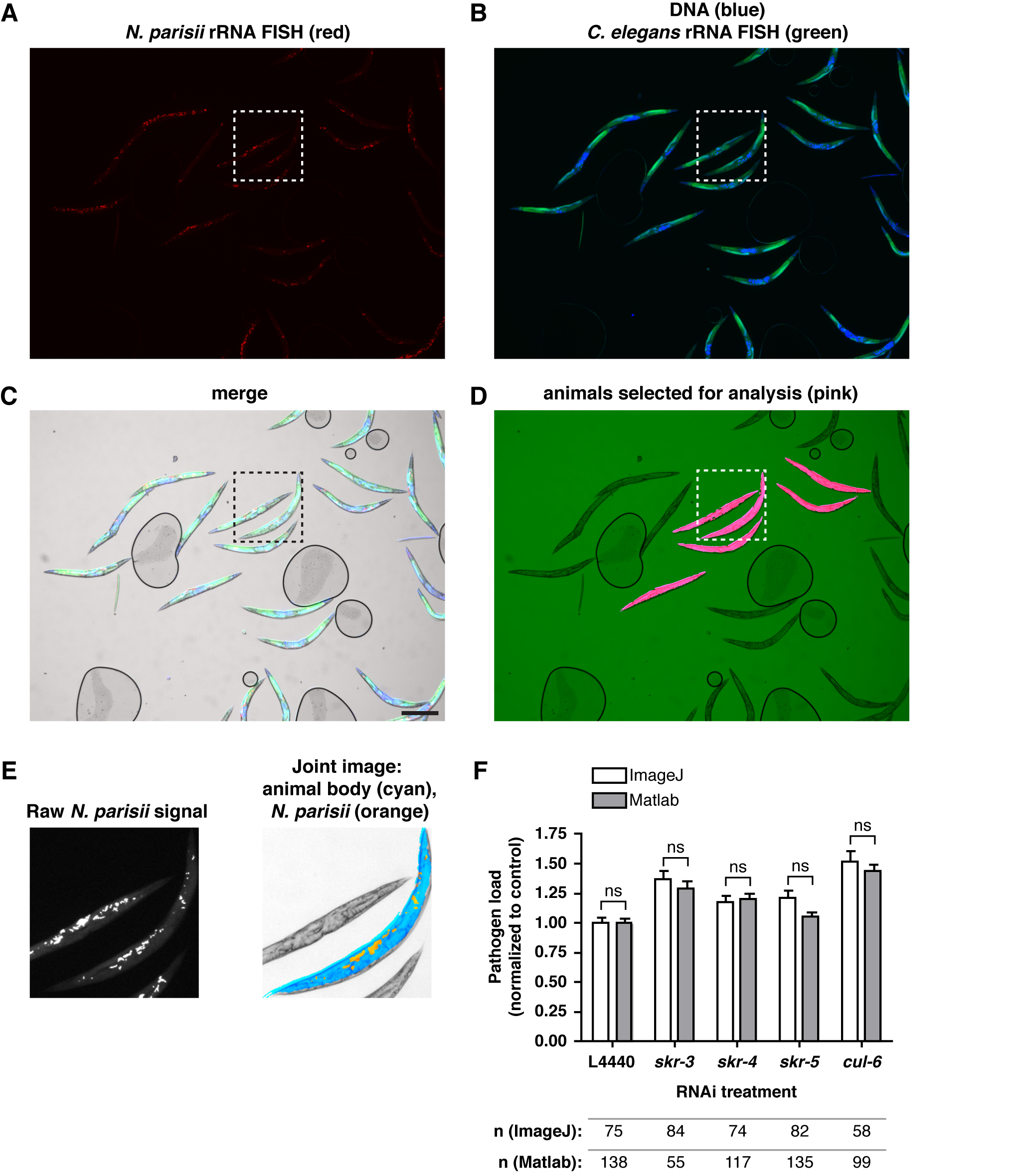

Supplement: Figure S3 — Comparison of ImageJ and Matlab methods of image analysis for pathogen load as measured by FISH. For automated Matlab analysis nematodes were fixed, stained with a FISH probe against N. parisii rRNA (red), a FISH probe against C. elegans rRNA (green), and DAPI for DNA (blue), and imaged using a 2.5× objective. DAPI and the C. elegans rRNA FISH probe signals were used to recognize each animal and the signal from the N. parisii FISH probe was used to determine the area of each animal occupied by the pathogen. Animals were manually censored for proper analysis by the software. A–E) Example of a single image analyzed by the Matlab program, showing the N. parisii FISH signal (A), the C. elegans rRNA (green) and DAPI (blue) signals (B), a merged image of all fluorescence and bright-field channels (C), and animals selected for analysis by the program (pink) (D). Note that animals touching bubbles, each other, or crossing the edges of the image, are not selected. E) An example of the analysis of a single infected nematode from a boxed in area in the panels above. The raw N. parisii signal (left panel), and a joint image generated by the program with areas recognized as the animal body (cyan) and the parasite (orange) (right panel) is shown. F) Animals from the same slides from a single experiment (one of independent experiments presented in Figure 2B) were imaged with a 10× objective and their pathogen load was measured with ImageJ, or they were imaged with the 2.5× objective and analyzed with the Matlab program. Mean pathogen area occupying each animal, normalized to mean L4440 control values +/− SEM, is shown. The number of animals analyzed for each condition (n) is indicated. Statistical significance was assessed using a two-way ANOVA with a Bonferroni posttest. While RNAi treatment significantly affected the results (p<0.0001), for each RNAi condition the analysis method did not yield statistically significant differences, p>0.05 (ns). (TIF) [file ppat.1004200.s003.tif]

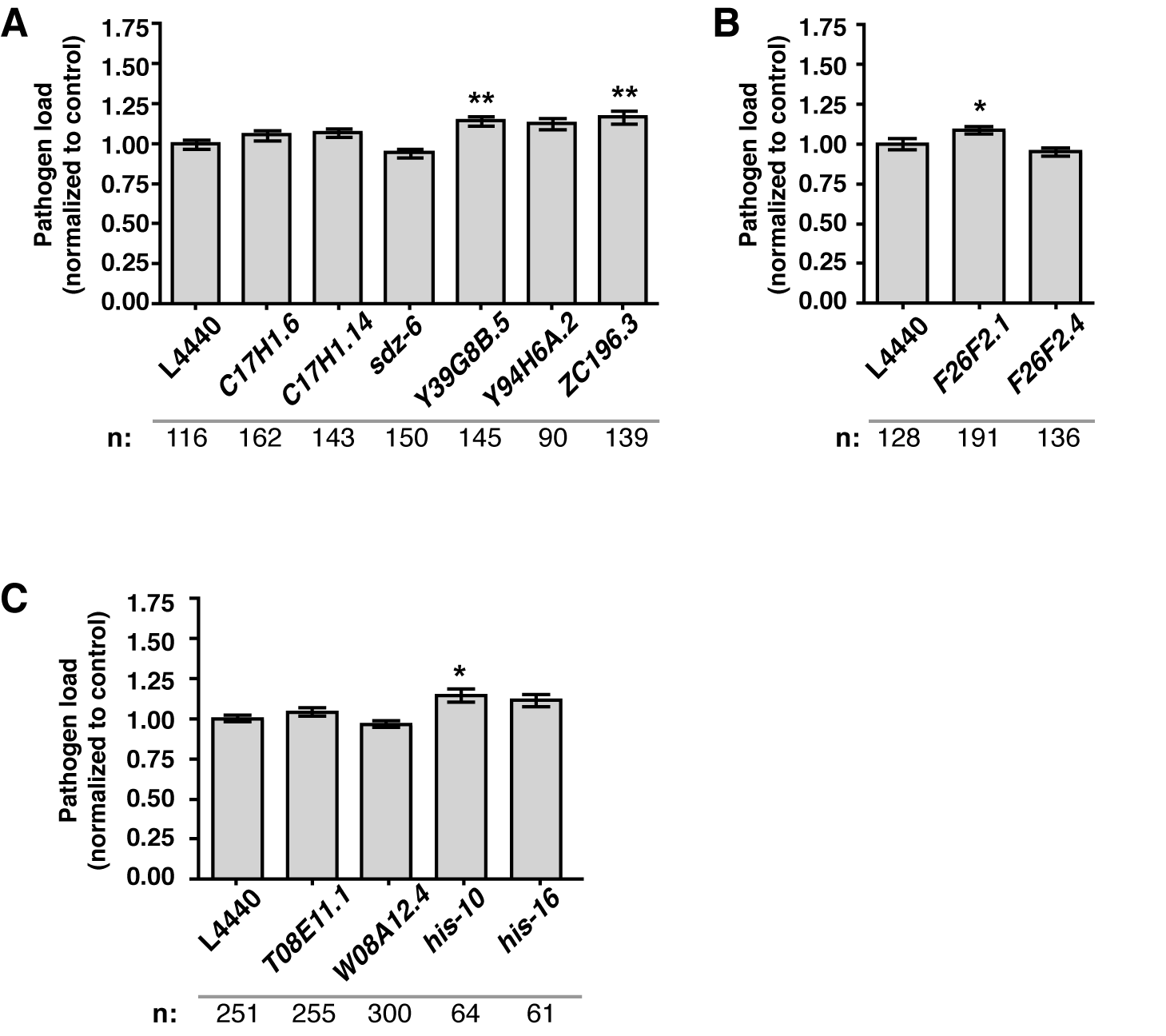

Supplement: Figure S4 — Analysis of pathogen load in animals treated with RNAi against genes upregulated during N. parisii infection. Quantification of pathogen load (see Materials and Methods) in nematodes treated with RNAi against the indicated genes. A, B) Synchronized animals were grown for two days on dsRNA-expressing bacteria. (C) Synchronized animals were grown for one day on OP50-1 E. coli followed by one day on dsRNA-expressing bacteria. Pathogen area occupying each RNAi-treated animal was normalized to mean L4440 control values. The number of animals analyzed for each condition (n) is indicated. Mean +/− SEM is shown for all analyzed animals (data are from at least one independent experiment comprised of two separate populations of animals). Statistical significance was assessed using a one-way ANOVA with Dunnett's Multiple Comparisons Test (**p<0.01, *p<0.05). (TIF) [file ppat.1004200.s004.tif]

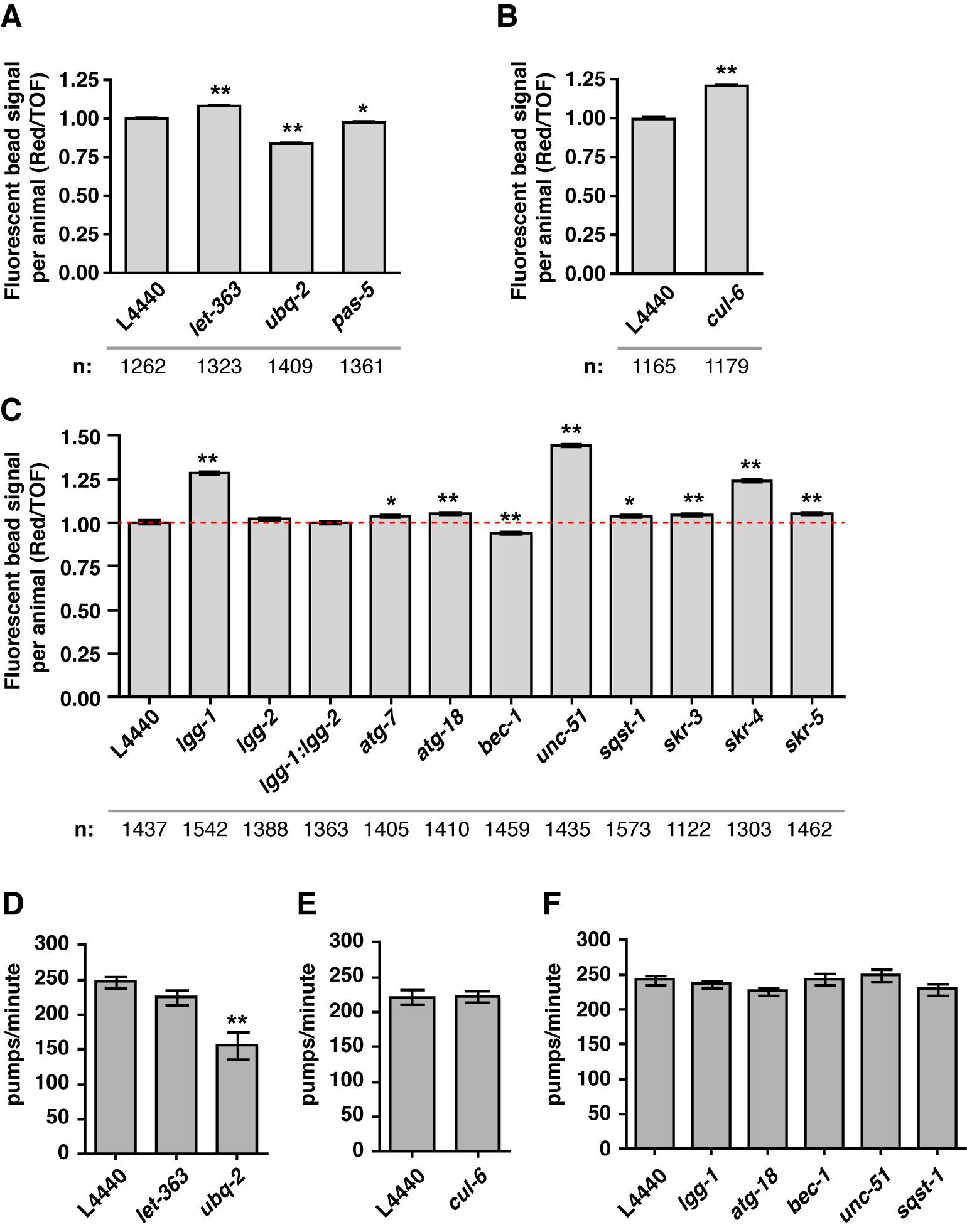

Supplement: Figure S5 — Analysis of animal feeding rates by quantification of fluorescent bead accumulation and pharyngeal pumping under different RNAi conditions. A–C) RNAi-treated animals were fed fluorescent beads mixed with N. parisii spores for 30 min, fixed with PFA, and the fluorescence of accumulated beads in each animal was measured using the worm sorter. Red fluorescence was normalized to animal size and mean L4440 control values. The number of animals analyzed for each condition (n) is indicated. Mean +/− SEM is shown (data are from two independent experiments comprised of two separate populations of animals). D–E) Quantification of pharyngeal pumping rates of animals grown on indicated RNAi clones (n = 10). Synchronized animals were grown for one day on OP50-1 E. coli followed by one day on dsRNA-expressing bacteria prior to analysis (A, D). Synchronized animals were grown for two days on dsRNA-expressing bacteria prior to analysis (B, C, E, F). Statistical significance was assessed using a one-way ANOVA with Dunnett's Multiple Comparisons Test (**p<0.01, *p<0.05). (TIF) [file ppat.1004200.s005.tif]

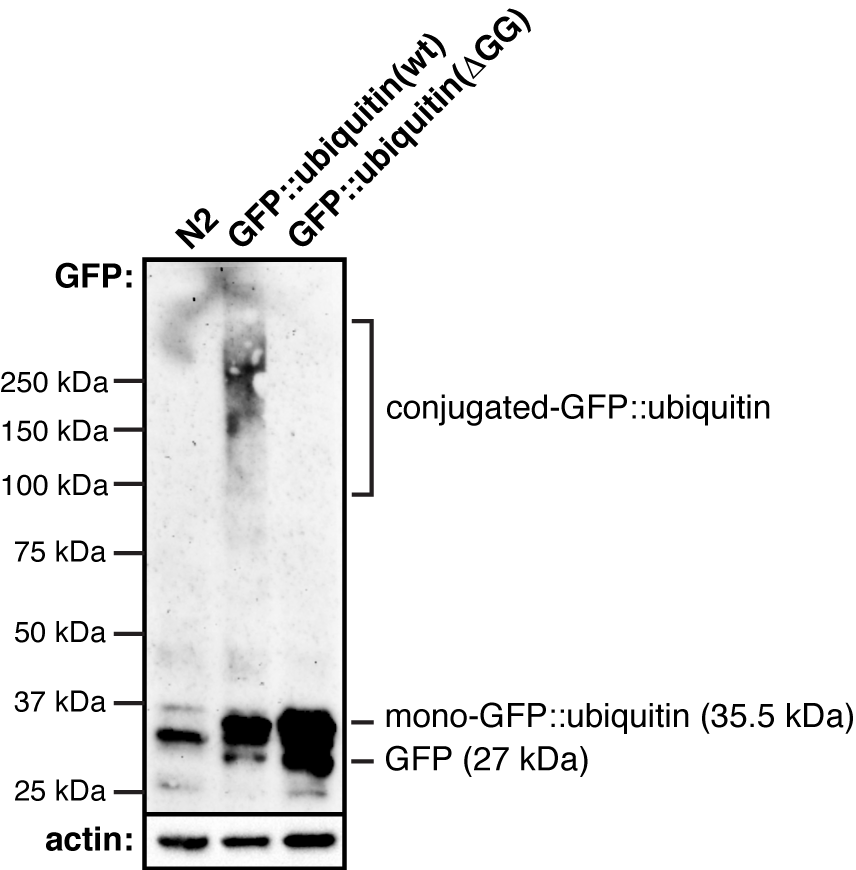

Supplement: Figure S6 — Western blot of C. elegans lysates indicate that GFP::ubiquitin fusion protein is conjugated onto substrates, while GFP::ubiquitinΔGG fusion protein is not. Lysates from equal numbers of N2 animals, and transgenic animals expressing wild-type GFP::ubiquitin, or conjugation-defective GFP::ubiquitinΔGG in their intestines were probed with anti-GFP antibody. The antibody recognized monomeric GFP::ubiquitin, free GFP, as well as GFP::ubiquitin conjugated to target proteins in the strain expressing wild-type GFP::ubiquitin (apparent as a high molecular weight smear) but not mutant GFP::ubiquitinΔGG. Proteins non-specifically recognized by the GFP antibody are seen in the N2 lysate. Anti-actin antibody was used as a loading control. (TIF) [file ppat.1004200.s006.tif]

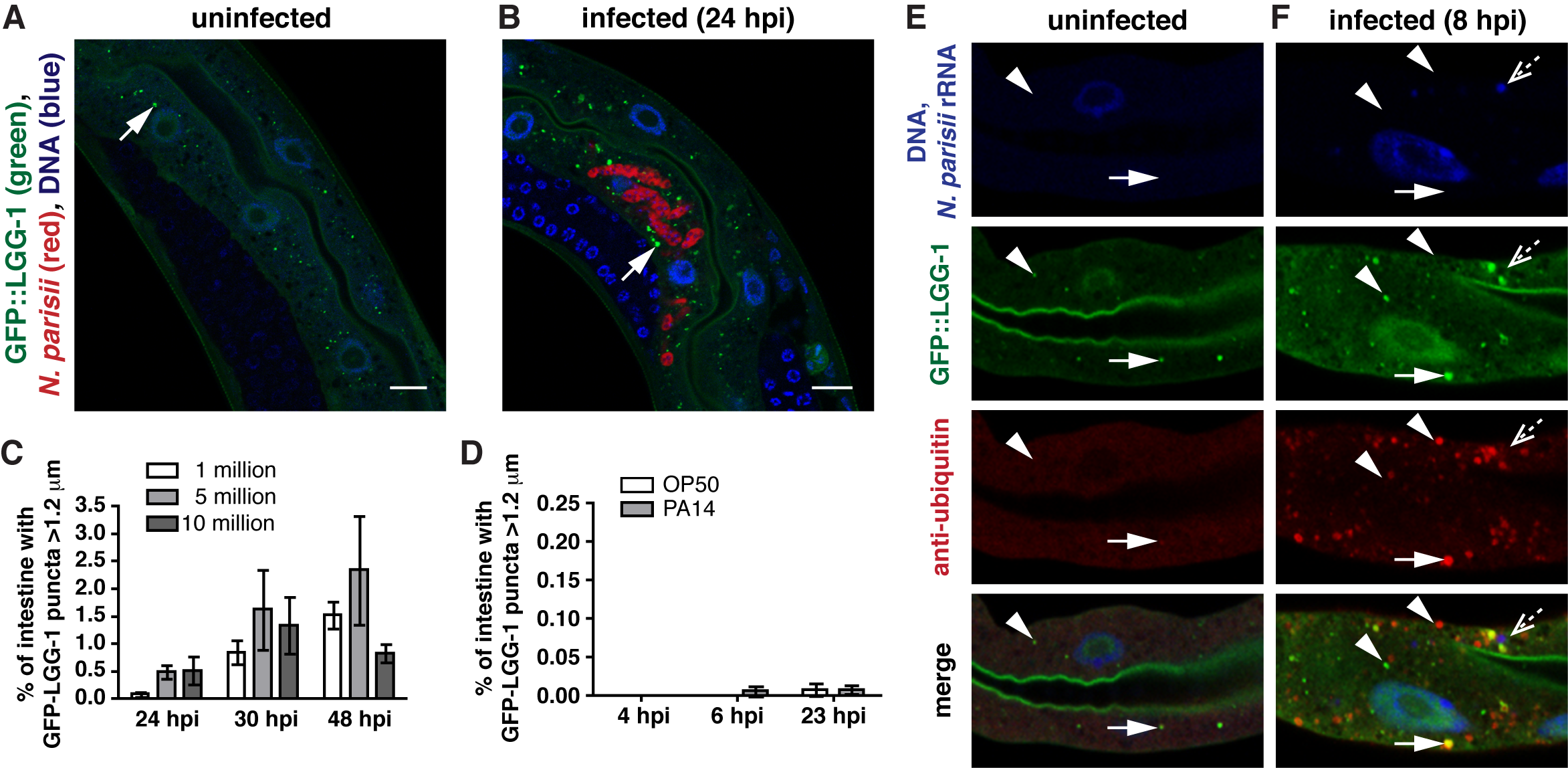

Supplement: Figure S7 — N. parisii infection causes clustering of the GFP::LGG-1 autophagy marker in the C. elegans intestine. (A–B) GFP::LGG-1-expressing transgenic animals were fixed and stained with a FISH probe against N. parisii rRNA (red) and DAPI for DNA (blue). A) Intestine of an uninfected nematode, and B) an N. parisii-infected nematode, 24 hpi, are shown. GFP::LGG-1 clusters are indicated with arrows. Scale bars = 10 µm. C) Quantification of GFP::LGG-1 clusters (see Materials and Methods) in animals infected with different doses of N. parisii spores at three different timepoints. For each condition, mean values from ten to twelve animals +/− SEM are shown. D) Quantification of GFP::LGG-1 clusters in live animals infected with Pseudomonas aeruginosa strain PA14, or grown on E. coli strain OP50 at three different timepoints. For each condition, mean values from four to eight animals +/− SEM are shown. E,F) GFP::LGG-1-expressing (green) transgenic animals were fixed and stained with FISH probes against N. parisii rRNA (blue), an anti-conjugated-ubiquitin antibody FK2 (red), and DRAQ5 for DNA (blue). Uninfected intestine (E) and infected intestine, 8 hpi (F), are shown. Scale bars = 10 µm. Conjugated-ubiquitin aggregates colocalizing with GFP::LGG-1 (arrow), or not colocalizing (arrowhead), and a N. parisii parasite cells (dashed arrow) are indicated. (TIF) [file ppat.1004200.s007.tif]

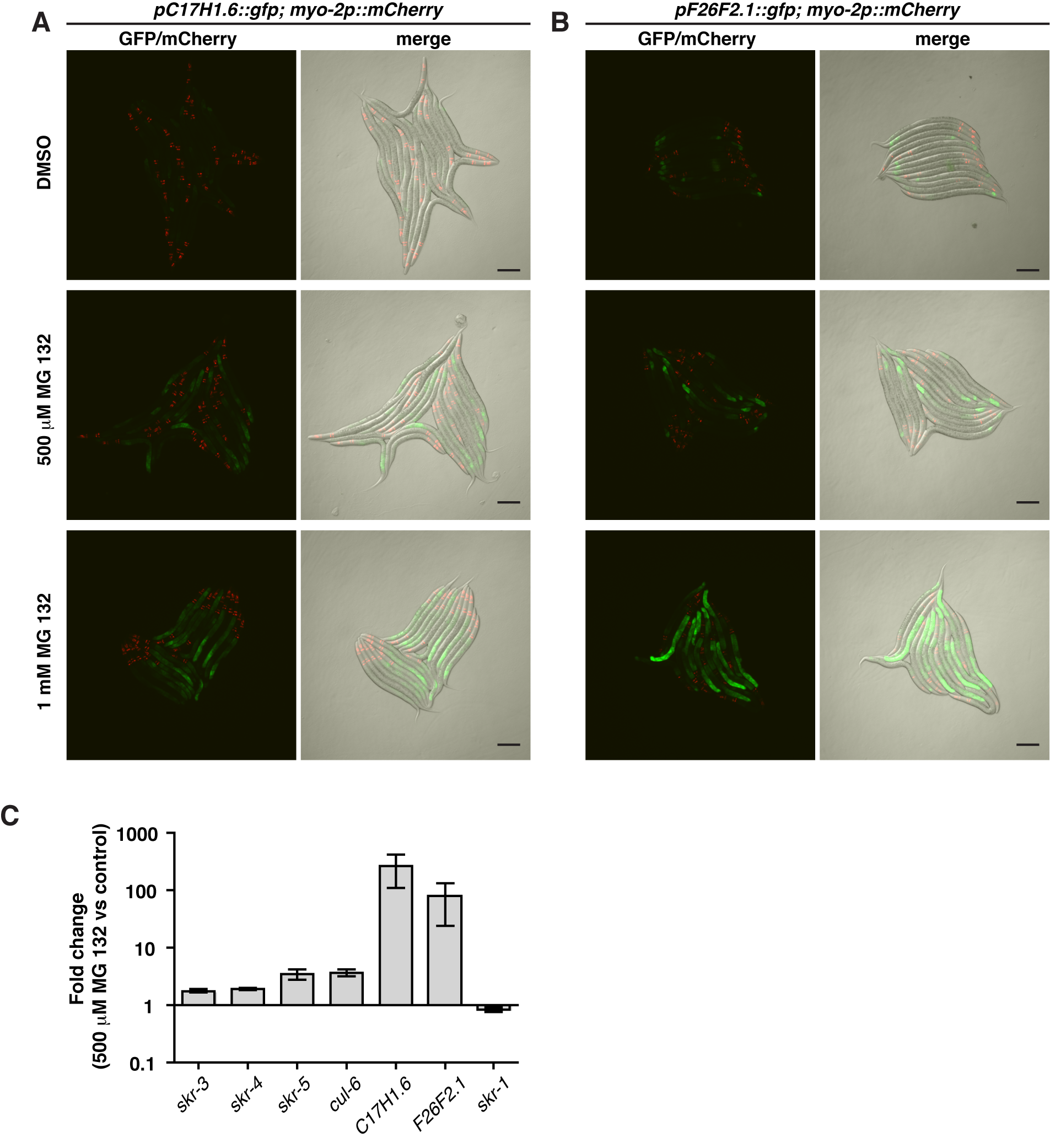

Supplement: Figure S8 — Pharmacological perturbation of the UPS induces infection response gene expression. A, B) Treatment with the proteasome inhibitor MG-132 induces expression of C17H1.6p::gfp (A) and F26F2.1p::gfp (B) in the absence of infection. Scale bars = 100 µm. C) MG-132 induces expression of endogenous mRNA transcripts for C17H1.6, F26F2.1, skr-3, skr-4, skr-5, cul-6, but not skr-1, as assessed by qRT-PCR. Mean +/− SEM of two independent experiments shown. (TIF) [file ppat.1004200.s008.tif]

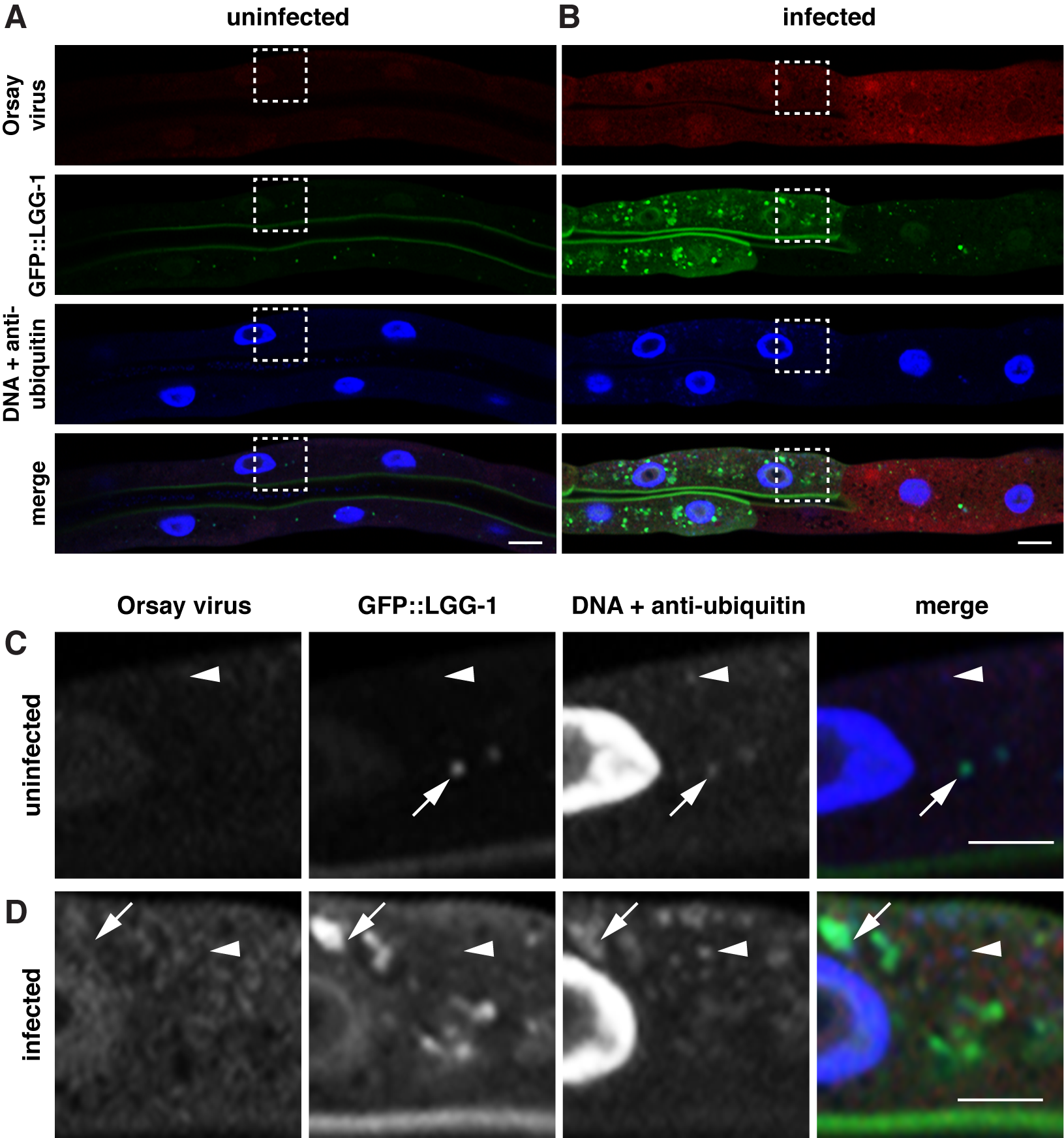

Supplement: Figure S9 — Infection with Orsay virus induces GFP::LGG-1 clusters. GFP::LGG-1-expressing (green) transgenic animals were fixed and stained with FISH probes against the Orsay virus RNA (red), an anti-conjugated-ubiquitin antibody FK2 (blue), and DAPI for DNA (blue). A) Uninfected intestine and B) Orsay virus-infected intestine, 24 hpi, are shown. Scale bars = 10 µm. C and D) Enlarged view of boxed in area from panels A and B, respectively, with conjugated-ubiquitin aggregates colocalizing with GFP::LGG-1 (arrow), or not colocalizing (arrowhead). Scale bars = 5 µm. (TIF) [file ppat.1004200.s009.tif]
