# Supplementary material for: Ubiquitin-Mediated Response to Microsporidia and Virus Infection in C. elegans
Source: PLoS Pathog. 2014 Jun 19;10(6):e1004200. doi: 10.1371/journal.ppat.1004200 (PMC4063957; doi:10.1371/journal.ppat.1004200)
Supplement: Text S1 — Supplemental text file describing: Microarray analysis of genes regulated by N. parisii infection; Comparisons between genes regulated by N. parisii and gene sets regulated by other pathogens and stressors; GO terms enriched at later timepoints of infection with N. parisii; Functional analysis of N. parisii-upregulated genes; Enrichment of F-box, FTH and MATH domains in genes regulated by N. parisii infection; The drugs fumagillin and FUdR limit microsporidia proliferation within C. elegans; Feeding controls for RNAi treatments that affect pathogen load; Supplemental Materials and Methods (Affymetrix microarray analysis; DAVID analysis; Automatic method for estimating pathogen load; Feeding assay; Pharyngeal pumping rates; GFP::LGG-1 imaging during N. parisii infection; Orsay virus, GFP::LGG-1, and conjugated-ubiquitin imaging; GFP::LGG-1 puncta quantification; Western blot analysis); and References. (DOCX) [file ppat.1004200.s019.docx]

**Supplemental Text**

**Ubiquitin-mediated response to microsporidia and virus infection in *C. elegans***

Malina A. Bakowski, Christopher A. Desjardins, Margery G. Smelkinson, Tiffany A. Dunbar, Isaac F. Lopez-Moyado, Scott A. Rifkin, Christina A. Cuomo, Emily R. Troemel.

**Microarray analysis of genes regulated by *N. parisii* infection**

To extend and confirm our RNA-seq analyses, we also performed full-genome Affymetrix microarray analysis of the response to *N. parisii* infection. In contrast to RNA-seq studies where sterile *fer-15;fem-1 C. elegans* were used to prevent internal hatching of progeny, we used wild-type fertile N2 animals for infection. We collected RNA from uninfected and infected animals at a 34 hpi timepoint, when animals were infected with meronts. These studies revealed a set of 83 genes upregulated by infection and 6 genes downregulated by infection (Table S3). There was substantial agreement between microarray and RNA-seq analysis, as 70 out of 83 of the upregulated and 6 out of 6 of the downregulated genes identified by microarray analysis also changed expression more than two-fold at 30 or 40 hpi according to our RNA-seq analyses (Table S3). These microarray experiments provide validation of our RNA-seq studies, and also indicate that similar gene expression changes are found in sterile as well as fertile animals upon infection.

**Comparisons between genes regulated by *N. parisii* and gene sets regulated by other pathogens and stressors**

We found that several classes of genes upregulated by infection with extracellular, bacterial pathogens that were downregulated by infection with *N. parisii*. For example, Pfam domains associated with secreted *C. elegans* anti-microbial response genes, such as ShK, CUB-like, CUB_2, and Lectin_C domains, were over-represented among downregulated genes at 30, 40, and 64 hpi (Table S7). These genes have been implicated in defense against several extracellular pathogens, such as *P. aeruginosa* and *S. aureus* [[1](#_ENREF_1)]. The downregulation of many secreted anti-microbials may be a specific response of *C. elegans* to *N. parisii* infection, as extracellular secretion of antimicrobial factors would likely not combat infection with *N. parisii*, which is a pathogen that appears to replicate in direct contact with the cytosol. Alternatively, downregulation of genes associated with the *C. elegans* response to bacterial infections could represent an anti-fungal response, as similar inverse correlations have been observed in response to the human fungal pathogen *Candida albicans* [[2](#_ENREF_2)].

In contrast to the *N. parisii*-upregulated genes, we found a strong positive correlation between *N. parisii*-downregulated genes at later timepoints (30, 40 and 64 hpi) and sets of genes downregulated in response to *P. aeruginosa* or *S. aureus*, genes regulated by known *C. elegans* immune pathways, and genes downregulated by other stressful conditions (Figure S2). Downregulation of genes that occurs in response to all of these stressors could represent a global shift of resources away from growth and toward insult-specific defense or stress resistance mechanisms. Accordingly, collagen (the building-block of the nematode cuticle and egg-shell) and VWD (von-Willebrand factor type D domain, which in *C. elegans* is found mainly in egg-yolk proteins called vitellogenins) domains were over-represented among down-regulated genes (Table S7). Genes implicated in detoxification and encoding the cytochrome p450 domain and the UDP-glucuronosyltransferase (UDPGT) domain were also significantly (p-value <0.05) downregulated at 40 and 64 hpi during infection (Table S7), which may reflect the more severe disruption of metabolic functions of the *C. elegans* intestine during late infection with *N. parisii*, or a specific suppression of host immunity by the parasite.

As mentioned above, more genes are downregulated during infection than upregulated (Figure 1C, Table S2). Correspondingly, a greater number of GO and KEGG terms were associated with downregulated genes. These were mostly related to metabolism and biosynthesis (Table S7). At 30 hpi cellular lipid catabolic processes were downregulated and at 40 and 64 hpi lipase activity, fatty acid metabolism and lysosome pathway components were also downregulated. The growth and spread of microsporidia meronts within the *C. elegans* intestine is associated with loss of gut granules of the infected animal [[3](#_ENREF_3)]. Therefore, lack of nutrients caused by the parasite burden and compromised intestine function may affect expression of host metabolic enzymes. However, microsporidia may also repress transcription of enzymes involved in host catabolism in an effort to reserve available pools of resources for their own consumption.

**GO terms enriched at later timepoints of infection with *N. parisii***

At 40 hpi infection-induced genes were enriched for nucleosome components (histones). Histones package DNA into heterochromatin, where gene transcription is generally repressed. As shown in Figure 1C, the number of significantly downregulated genes increases over seven-fold, from 26 genes at 30 hpi to 185 genes at 40 hpi, and we speculate this downregulation may be related to increased expression of histones at 40 hpi. Alternatively, histones were previously associated with anti-microbial activity [[4](#_ENREF_4),[5](#_ENREF_5)] and may directly participate in the *C. elegans* defense response during microsporidia infection. Finally, at 64 hpi, upregulated genes are enriched for the defense response, cell-substrate adhesion, and intermediate filament cytoskeleton associated terms. At this timepoint, infected *C. elegans* intestinal cells are full of microsporidia spores, which are exiting the infected tissue. Therefore, increased expression of cell-substrate adhesion and intermediate filament associated genes could improve structural integrity of the infected organ.

**Functional analysis of *N. parisii*-upregulated genes**

We determined *N. parisii* pathogen load in animals treated with RNAi against a number of genes that were significantly upregulated during infection. We examined genes that were very highly induced early during infection (*C17H1.6*, *C17H1.14*, *F26F2.1*, *F26F2.4*, *Y39G8B.5*, *sdz-6*), or belonged to one of the enriched gene classes identified through GO term and Pfam domain analysis (Table 1) (positive regulation of growth: *T08E11.1, W08A12.4;* DUF713 domain-containing: *ZC196.3;* DUF684 domain-containing: *Y94H6A.2*; histones: *his-10*, *his-16*)*.* Out of all the genes tested we observed a significant but small (8.5 to 17%) increase in pathogen load upon knockdown of four genes: *Y39G8B.5, ZC196.3, F26F2.1,* or *his-10* (Figure S2G-I). Interestingly, *Y39G8B.5* shares sequence similarity with the *C. elegans* *ikke-1*, a homolog of the inhibitor of NFκB kinase ε subunit.

**Enrichment of F-box, FTH and MATH domains in genes regulated by *N. parisii* infection**

F-box proteins are responsible for recruiting target proteins to SCF (Skp1-Cul1-F-box) multi-subunit E3 ubiquitin ligase complexes, which transfer ubiquitin to these substrates. F-box proteins interact with the SCF complex via their F-box domains and use a variety of domains to interact with their target substrates. In *C. elegans,* FTH domains have been hypothesized to act as such substrate recognition domains [[6](#_ENREF_6)] and, with one exception where only the FTH domain was present, upregulated genes in our analysis that encoded for an FTH domain also encoded for an F-box domain, suggesting that the encoded proteins can potentially recognize a target substrate, and also recruit ubiquitin machinery.

MATH domains are thought to act analogously to the FTH domain in multisubunit E3 ubiquitin ligase complexes when paired with a BTB domain, where the BTB domain interacts directly with the cullin scaffold of the complex. However, the MATH domain-containing genes that were significantly upregulated during *N. parisii* infection did not encode for obvious BTB domains. These genes encoded only for a variable number of MATH domains (from two to six) and in one case for a MATH domain and an F-box-related F-box_2 domain. Therefore, MATH domain-encoding genes upregulated during infection may act through E3 ligase- and ubiquitin-independent output mechanisms.

**The drugs fumagillin and FUdR limit microsporidia proliferation within *C. elegans***

Fumagillin is an anti-microsporidia drug that can block microsporidia growth in several different hosts [[7](#_ENREF_7),[8](#_ENREF_8),[9](#_ENREF_9)], and we found that fumagillin could also block *N. parisii* growth in *C. elegans* (Figure 2D). The mechanism of action of fumagillin is fairly well-established, because it has been investigated as a drug for anti-cancer treatment due to its ability to inhibit angiogenesis [[10](#_ENREF_10)]. Fumagillin covalently binds and inhibits methionine aminopeptidase2 (MAP-2), an enzyme that catalyzes the hydrolytic removal of N-terminal methionine residues from nascent proteins [[11](#_ENREF_11)]. The *C. elegans* *map-2* gene encodes a methionine aminopeptidase2 ortholog that is sensitive to fumagillin, but pharmacological inhibition of MAP-2 by feeding or injection of a related antibiotic (ovalicin) had no phenotypic effects on *C. elegans* [[12](#_ENREF_12)]. However, RNAi mediated knockdown of *map-2* in *C. elegans* did lead to sterility due to germline proliferation defects [[12](#_ENREF_12)]. In our experiments we administered fumagillin by feeding and measured pathogen load in a conditionally sterile strain of *C. elegans* (see Materials and Methods), and thus the sterilizing effect of fumagillin should not have an impact on our infection assays. While there may be other effects of fumagillin on the host that have not yet been described, we believe that the simplest explanation for fumagillin inhibition of *N. parisii* growth is that it directly affects the pathogen physiology.

We also used Floxuridine (5-fluorodeoxyuridine or FUdR) as an anti-microsporidia drug. FUdR is a fluorinated pyrimidine that is an anti-cancer drug, working primarily by preventing the generation of thymidine that is needed for DNA synthesis. It is toxic to rapidly replicating cells and is commonly used in *C. elegans* longevity and pathogen (e.g. *Pseudomonas aeruginosa*) survival studies to sterilize animals. Because *N. parisii* encodes for the primary target of FUdR, the thymidylate kinase, and replicates very rapidly in infected *C. elegans* intestines [[13](#_ENREF_13)], we speculated that FUdR, by inhibiting DNA synthesis of these pathogens, would prevent their reproduction. Indeed, treatment of infected animals with FUdR significantly inhibited *N. parisii* replication (Figure 2E). It is important to note that FUdR treatment has been found to promote improved proteostasis and increase resistance to heat stress in *C. elegans* [[14](#_ENREF_14),[15](#_ENREF_15)]. However, studies showing increased proteostasis caused by FUdR used 1.7-fold higher FUdR concentrations than those used in our studies, but most importantly also observed an approximately 20% increase in resistance to heat stress following the equivalent time of FUdR treatment, compared to the drastic 95% inhibition of microsporidia replication we observe in our study (Figure 2E). Thus, we believe that the predominant mechanism by which FUdR blocks *N. parisii* growth in our assays is through direct effects on pathogen physiology, although it is possible that it may also increase host resistance to infection.

**Feeding controls for RNAi treatments that affect pathogen load**

As stated in the main text, we performed feeding controls using fluorescent beads to assess whether animals with altered pathogen load had altered accumulation of beads in their intestinal lumen. Almost all RNAi treatments that caused increased pathogen load did not cause increased accumulation of beads in the lumen. However, RNAi against *cul-6* and *lgg-1* did cause slightly increased levels of beads to accumulate in the intestine (Fig. S5B, C). This result was somewhat surprising, as RNAi against *lgg-1* and other autophagy components have previously been shown not to affect feeding or defecation rate. Indeed, we confirmed that feeding rate, while significantly decreased by *ubq-2* RNAi (Fig. S5D), was not altered by *lgg-1* or *cul-6* RNAi (Fig. S5E, F). Therefore, *lgg-1* and *cul-6* RNAi may somehow alter the transit time of contents through the *C. elegans* lumen, making it formally possible that the increased pathogen load in these RNAi-treated animals is due to an increased inoculum. However, the fact that other RNAi treatments also caused increased accumulation of fluorescent beads but did not cause increased pathogen load (e.g. *unc-51* and *skr-4* RNAi), suggests that simply having more initial inoculum does not necessarily lead to increased pathogen load, and that the level of initial inoculum may not be a rate-limiting factor in pathogen infection of wild-type animals.

**Supplemental Materials and Methods**

Affymetrix microarray analysis

Arrested N2 wild-type L1 animals were placed onto 6 cm NGM plates seeded with *E. coli* OP50 and grown until the L3/L4 stage, and then infected with an *N. parisii* spore prep or treated with control diluent. Infections were allowed to proceed for 33-34 h, when approximately half of the animals displayed meronts. Animals were then harvested for RNA extraction as described [[16](#_ENREF_16)]. Three independent replicates of each treatment were performed. RNA samples were prepared and hybridized on Affymetrix full-genome GeneChips for *C. elegans* at the Veterans Medical Research Foundation Microarray and NGS core, hosted at the VA San Diego Healthcare System, according to instructions from Affymetrix (<http://www.affymetrix.com>). Differential expression analysis was performed with VAMPIRE software using Bayesian statistical test to identify gene expression differences between infected and uninfected samples that were statistically significant [[17](#_ENREF_17)].

DAVID analysis

The Database for Annotation, Visualization and Integrated Discovery (DAVID) v6.7 Functional Annotation tool was used to perform enrichment analysis for GO, KEGG, and Pfam domains among our significantly regulated genes [14,15]. To eliminate redundancy, each term had at least 30% of associated genes not associated with any other term with a more significant p-value.

Automatic method for estimating pathogen load

In addition to the semi-manual method using ImageJ, we also used a custom fully automatic method for estimating pathogen load written in Matlab v2012b (see:http://talmai-oliveira.blogspot.com/2011/06/how-to-cite-matlab-bibtex-latex.html). The animals were grown, infected and processed as described above, except they were also stained with a FISH probe specific to rRNA of *C. elegans* (Ce966 (5’-GGATAGCTCCTCGGCAGG), conjugated to FAM (fluorescein) (Biosearch)). Animals were imaged using a Zeiss AxioImager microscope with a 2.5X objective. We calculated pathogen load in two steps. First we identified pixels belonging to individual worms. Then we segmented the worms into infected and uninfected areas. To distinguish worms from background, we first detected edges in the *C. elegans* RNA FISH and DAPI images using built-in Laplacian of Gaussian and Canny edge detection algorithms. We took the union of these edge images, morphologically closed gaps in the border, filled in the interior of objects, and eliminated small objects as well as objects that touched the image boundary so that we would only work with complete worms. This generated a set of segmentation masks identifying pixels belonging to individual worms. We then identified infected areas by masking the non-worm areas of the *N. parisii* rRNA FISH image using the segmentation masks from the first step. Working with one worm at a time, we fit two Gaussians to the pixel intensities of this masked image and used these to separate the pixels into two classes: bright ones marked pathogen staining while the dimmer distribution represented background staining in the worm. We estimated pathogen load as the fraction of pathogen pixels out of all the worm pixels.

Feeding assay

Synchronized, RNAi-treated animals were fed a mixture of 2 million spores and 5 μL of Fluoresbrite® Polychromatic Red Microspheres (Polysciences, Inc.) in a total volume of 250 uL per 6 cm plate. After 30 min of incubation at 25^o^C, plates were placed on ice, animals washed off with ice-cold PBS-T, and immediately fixed with 4% PFA. Red fluorescence signal in fixed worms was analyzed using the COPAS biosorter (Union Biometrica) [[18](#_ENREF_18)].

Pharyngeal pumping rates

Synchronized L1 animals were grown for 2 days on the indicated RNAi bacteria at 25^o^C, except for animals grown on *ubq-2* and *let-363* RNAi and respective control, which were grown for 1 day on OP50-1 *E. coli* prior to transfer to RNAi for 1 day in order to limit developmental defects. Pharyngeal pumping rates were measured under a dissecting scope in 1 min intervals and pumping rate (pumps/minute) were determined in 10 animals per condition. Prior to measurements being taken, animals were incubated at room temperature (22°C) for 1 h.

GFP::LGG-1 imaging during *N. parisii* infection

Synchronized DA2123 *adIs2122[lgg-1p::gfp::lgg-1]* animals were grown for 2 days at 25^o^C followed by infection with 10 million *N. parisii* spores. At 8 hpi, the infected animals were anesthetized with 10 mM levamisole, their intestines dissected out, and fixed for 15-30 min in 4% PFA. The intestines were stained with MicroB FISH probe against *N. parisii* rRNA, mounted in Vectashield with DAPI (Vector Laboratories) and imaged using a laser scanning confocal microscope with a 40x oil immersion objective (Zeiss LSM 700). For conjugated ubiquitin and GFP::LGG-1 colocalization experiment, FISH-stained intestines were also stained with FK2 anti-conjugated-ubiquitin antibody, the Alexa405 goat anti-mouse IgG (Molecular Probes) secondary antibody, and incubated for 10 min at room temperature with 5 μM DRAQ5 (Cell Signaling) in PBS-T prior to imaging. Stained intestines were mounted and imaged as described above.

Orsay virus, GFP::LGG-1, and conjugated-ubiquitin imaging

Synchronized DA2123 *adIs2122[lgg-1p::gfp::lgg-1]* animals were grown for 2 days at 25^o^C followed by infection with the Orsay virus. At 24 hpi, the infected animals were anesthetized with 10 mM levamisole, their intestines dissected out, and fixed for 15-30 min in 4% PFA. The intestines were then stained with FISH probes specific to Orsay RNA1 and RNA2 (Orsay1 (5’-GACATATGTGATGCCGAGAC), and Orsay2 (5’-GTAGTGTCATTGTAGGCAGC)) conjugated to CAL Fluor Red 610 (Biosearch). Staining with a mixture of both probes (each at 5 ng/uL) was performed as described for the *N. parisii* MicroB FISH probe, followed by staining with FK2 anti-conjugated-ubiquitin antibody and the Alexa405 goat anti-mouse IgG (Molecular Probes) secondary antibody. Stained intestines were mounted and imaged as described above.

GFP::LGG-1 puncta quantification

Synchronized DA2123 *adIs2122[lgg-1p::gfp::lgg-1]* animals were grown for 1 day at 20^o^C. The next day animals were infected with 1, 5, or 10 million spores and moved to 25^o^C. At indicated timepoints animals were fixed with 4% PFA and stained with MicroB probe as described above. For PA14 infections, animals were infected as described [[19](#_ENREF_19)]. Briefly, synchronized animals were grown for 1 day at 20^o^C. The next day the animals were transferred onto PA14 or OP50 seeded plates that have been incubated at 25^o^C for 48 h. Live animals were imaged at indicated timepoints and number of puncta >1.2 μm in diameter in randomly chosen intestine sections were quantified using ImageJ software.

Western blot analysis

Protein extracts from equal numbers of synchronized N2, ERT261, and ERT264 adult animals grown at 20^o^C for 3 days were prepared by lysis in Laemmli buffer, separated in 4-20% SDS-PAGE gels (BioRad), and transferred to PVDF membrane. Monoclonal antibodies against GFP (mix of clones 7.1 and 13.1, Roche), actin (clone JLA20, Calbiochem), and a goat anti-murine HRP as a secondary antibody (Calbiochem) were used to probe the blots. Immunoreactive proteins were visualized using chemiluminescence and signals were captured electronically with the ChemiDoc XRS+ system (BioRad).

**References:**

1. Simonsen KT, Gallego SF, Faergeman NJ, Kallipolitis BH (2012) Strength in numbers: "Omics" studies of C. elegans innate immunity. Virulence 3: 477-484.

2. Pukkila-Worley R, Ausubel FM, Mylonakis E (2011) Candida albicans infection of Caenorhabditis elegans induces antifungal immune defenses. PLoS pathogens 7: e1002074.

3. Troemel ER, Felix MA, Whiteman NK, Barriere A, Ausubel FM (2008) Microsporidia are natural intracellular parasites of the nematode *Caenorhabditis elegans*. PLoS Biol 6: 2736-2752.

4. Cho JH, Park IY, Kim HS, Lee WT, Kim MS, et al. (2002) Cathepsin D produces antimicrobial peptide parasin I from histone H2A in the skin mucosa of fish. FASEB journal : official publication of the Federation of American Societies for Experimental Biology 16: 429-431.

5. Kawasaki H, Iwamuro S (2008) Potential roles of histones in host defense as antimicrobial agents. Infectious disorders drug targets 8: 195-205.

6. Thomas JH (2006) Adaptive evolution in two large families of ubiquitin-ligase adapters in nematodes and plants. Genome Res 16: 1017-1030.

7. Didier ES, Maddry JA, Brindley PJ, Stovall ME, Didier PJ (2005) Therapeutic strategies for human microsporidia infections. Expert review of anti-infective therapy 3: 419-434.

8. Didier PJ, Phillips JN, Kuebler DJ, Nasr M, Brindley PJ, et al. (2006) Antimicrosporidial activities of fumagillin, TNP-470, ovalicin, and ovalicin derivatives in vitro and in vivo. Antimicrobial agents and chemotherapy 50: 2146-2155.

9. Williams GR, Sampson MA, Shutler D, Rogers RE (2008) Does fumagillin control the recently detected invasive parasite Nosema ceranae in western honey bees (Apis mellifera)? Journal of invertebrate pathology 99: 342-344.

10. Yin SQ, Wang JJ, Zhang CM, Liu ZP (2012) The development of MetAP-2 inhibitors in cancer treatment. Current medicinal chemistry 19: 1021-1035.

11. Sin N, Meng L, Wang MQ, Wen JJ, Bornmann WG, et al. (1997) The anti-angiogenic agent fumagillin covalently binds and inhibits the methionine aminopeptidase, MetAP-2. Proceedings of the National Academy of Sciences of the United States of America 94: 6099-6103.

12. Boxem M, Tsai CW, Zhang Y, Saito RM, Liu JO (2004) The C. elegans methionine aminopeptidase 2 analog map-2 is required for germ cell proliferation. FEBS letters 576: 245-250.

13. Cuomo CA, Desjardins CA, Bakowski MA, Goldberg J, Ma AT, et al. (2012) Microsporidian genome analysis reveals evolutionary strategies for obligate intracellular growth. Genome research 22: 2478-2488.

14. Feldman N, Kosolapov L, Ben-Zvi A (2014) Fluorodeoxyuridine Improves Caenorhabditis elegans Proteostasis Independent of Reproduction Onset. PLoS One 9: e85964.

15. Angeli S, Klang I, Sivapatham R, Mark K, Zucker D, et al. (2013) A DNA synthesis inhibitor is protective against proteotoxic stressors via modulation of fertility pathways in Caenorhabditis elegans. Aging 5: 759-769.

16. Troemel ER, Chu SW, Reinke V, Lee SS, Ausubel FM, et al. (2006) p38 MAPK regulates expression of immune response genes and contributes to longevity in C. elegans. PLoS genetics 2: e183.

17. Hsiao A, Worrall DS, Olefsky JM, Subramaniam S (2004) Variance-modeled posterior inference of microarray data: detecting gene-expression changes in 3T3-L1 adipocytes. Bioinformatics 20: 3108-3127.

18. Pulak R (2006) Techniques for analysis, sorting, and dispensing of C. elegans on the COPAS flow-sorting system. Methods in molecular biology 351: 275-286.

19. Powell JR, Ausubel FM (2008) Models of Caenorhabditis elegans infection by bacterial and fungal pathogens. Methods in molecular biology 415: 403-427.
